# Hospital Strain During the COVID-19 Pandemic and Outcomes in Older Racial and Ethnic Minority Adults

**DOI:** 10.1001/jamanetworkopen.2024.38563

**Published:** 2024-10-15

**Authors:** Laurent G. Glance, Karen E. Joynt Maddox, Patricia W. Stone, E. Yoko Furuya, Jingjing Shang, Mark J. Sorbero, Ashley Chastain, Stewart J. Lustik, Andrew W. Dick

**Affiliations:** 1 Department of Anesthesiology and Perioperative Medicine, University of Rochester School of Medicine, Rochester, New York; 2 Department of Public Health Sciences, University of Rochester School of Medicine, Rochester, New York; 3 RAND Health, RAND, Boston, Massachusetts; 4 Department of Medicine, Washington University in St Louis, St Louis, Missouri; 5 Center for Health Economics and Policy at the Institute for Public Health, Washington University in St Louis, St Louis, Missouri; 6 Columbia School of Nursing, Center for Health Policy, New York, New York; 7 Department of Medicine, Division of Infectious Diseases, Columbia University Irving Medical Center, New York, New York

## Abstract

**Importance:**

Marginalized populations have been disproportionately affected by the COVID-19 pandemic. Critically ill patients belonging to racial and ethnic minority populations treated in hospitals operating under crisis or near-crisis conditions may have experienced worse outcomes than White individuals.

**Objective:**

To examine whether hospital strain was associated with worse outcomes for older patients hospitalized with sepsis and whether these increases in poor outcomes were greater for members of racial and ethnic minority groups compared with White individuals.

**Design, Setting, and Participants:**

In this cross-sectional study, multivariable regression analysis was conducted to assess differential changes in all-cause 30-day mortality and major morbidity among older racial and ethnic minoritized individuals hospitalized with sepsis compared with White individuals and changes in hospital strain using Medicare claims data. Data were obtained on patients hospitalized between January 1, 2016, and December 31, 2021, and analyzed between December 16, 2023, and July 11, 2024.

**Exposure:**

Time-varying weekly hospital percentage of inpatients with COVID-19.

**Main Outcomes and Measures:**

Composite of all-cause 30-day mortality and major morbidity.

**Results:**

Among the 5 899 869 hospitalizations for sepsis (51.5% women; mean [SD] age, 78.2 [8.8] years), there were 177 864 (3.0%) Asian, 664 648 (11.3%) Black, 522 964 (8.9%) Hispanic, and 4 534 393 (76.9%) White individuals. During weeks when the hospital COVID-19 burden was greater than 40%, the risk of death or major morbidity increased nearly 2-fold (adjusted odds ratio [AOR], 1.90; 95% CI, 1.80-2.00; *P* < .001) for White individuals compared with before the pandemic. Asian, Black, and Hispanic individuals experienced 44% (AOR, 1.44; 95% CI, 1.28-1.61; *P* < .001), 21% (AOR, 1.21; 95% CI, 1.11-1.33; *P* < .001), and 45% (AOR, 1.45; 95% CI, 1.32-1.59; *P* < .001) higher risk of death or morbidity, respectively, compared with White individuals when the hospital weekly COVID-19 burden was greater than 40%.

**Conclusion and Relevance:**

In this cross-sectional study, older adults hospitalized with sepsis were more likely to die or experience major morbidity as the hospital COVID-19 burden increased. These increases in adverse outcomes were greater in magnitude among members of minority populations than for White individuals.

## Key Points

**Question:**

Was hospital strain during the COVID-19 pandemic associated with outcomes among older adults of racial and ethnic minority groups hospitalized with sepsis compared with White individuals?

**Findings:**

In this cross-sectional study including more than 5 million older patients (mean age, 78.2 years), White individuals experienced a 90% increased risk of adverse outcomes when the hospital COVID-19 burden was greater than 40%. Asian, Black, and Hispanic individuals experienced 44%, 21%, and 45% higher risk of adverse outcomes than White individuals during these levels of hospital strain, respectively.

**Meaning:**

The findings of this study suggest that older adults hospitalized with sepsis in hospitals under severe stress were more likely to experience adverse outcomes as the hospital COVID-19 burden increased, and these increases were greater among racial and ethnic minoritized individuals.

## Teaser

This cross-sectional study examines morbidity and mortality in older racially and ethnically minoritized patients with sepsis who were hospitalized during the COVID-19 pandemic at various states of hospital burden.

## Introduction

More than 1.7 million US individuals develop sepsis each year, and one-third of hospital deaths are attributable to sepsis.^1^ In the Medicare population alone, the annual cost of sepsis is estimated to be more than $57 billion.^2^ Furthermore, there are reports of major racial and ethnic disparities in sepsis-related mortality rates, with American Indian or Alaska Native, Black, and Hispanic populations having markedly higher mortality rates associated with this condition compared with White populations.^3,4,5,6,7,8^ Despite the fact that nearly 4 decades have passed since the Department of Health and Human Services first reported that Black individuals had 60 000 excess deaths compared with White individuals each year,^9^ disparities remain prevalent. Recent estimates report 1.6 million excess deaths and 80 million excess years of life lost in the Black population compared with the White population over the past 22 years.^10^ Sepsis may be an important factor in these differences.^3,7^

The care of patients with sepsis is resource intensive, and hospital strain may unmask the results of latent hospital-level causes of disparities among the most critically ill patients. During the COVID-19 pandemic, enormous strain was placed on the health care system, including shortages of inpatient and intensive care unit beds, overworked staff, staff burnout, changes in staffing levels, use of nontraditional staffing, inadequate access to personal protective equipment, and decisions to withhold or reallocate lifesaving interventions under crisis conditions.^11,12,13,14,15,16,17^ Prior work found that hospitals under stress during the pandemic experienced higher mortality rates for patients admitted with acute myocardial infarctions,^18^ strokes,^19^ or for elective and nonelective surgery.^20^ However, no strong evidence that these changes were greater in racial and ethnic minoritized individuals than in White individuals was observed.

It is crucial to understand the interaction between hospital strain and health inequities, particularly as hospital closures in rural areas and high occupancy rates in urban hospitals threaten to increase strain on an ongoing basis.^21^ Using national Medicare data, this study therefore addressed 2 issues. The first was whether patients admitted with sepsis had higher rates of mortality and morbidity in hospitals under severe stress, quantified using the weekly percentage of patients hospitalized with COVID-19. The second was whether these increases in adverse outcomes were greater in individuals belonging to racial and ethnic minority groups compared with White individuals. Understanding how hospitals respond to severe stress when treating critically ill patients may help inform policies that promote equitable care in future public health crises.

## Methods

This study was approved by the Columbia University Institutional Review Board, which determined that informed consent was not necessary because it was exempt research. This study was covered by a data use agreement with the Centers for Medicare & Medicaid Services (CMS). The Strengthening the Reporting of Observational Studies in Epidemiology (STROBE) reporting guideline was used to guide the reporting of this study.^22^

### Data Source

This retrospective cross-sectional analysis used patient-level data from the 100% Medicare Provider Analysis and Review File and the Master Beneficiary Summary file between 2016 and 2021. These databases include demographic information, including age, sex, self-reported race and ethnicity (Asian or Other Pacific Islander [hereafter referred to as Asian], Black, Hispanic, North American Native, White, and other), captured at the time of Social Security enrollment; *International Statistical Classification of Disease and Related Health Problems, Tenth Revision* (*ICD-10*) diagnosis and procedure codes; date of admission; discharge destination; date of death; and hospital identifiers. These data were merged with the CMS Impact File, which included hospital characteristics (number of beds, average daily census, resident-to-bed ratio, disproportionate share hospital percentage, and geographic region).

### Study Population

**Figure 1.**
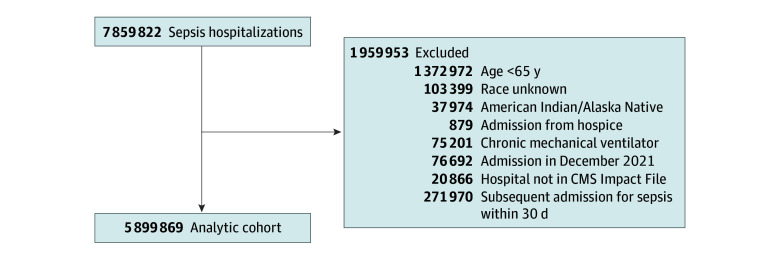
Study Flow Diagram for Analytic Cohort CMS indicates Centers for Medicare & Medicaid Services.

We identified 7 859 822 hospitalizations for sepsis between January 1, 2016, and December 31, 2021. We identified all cases of sepsis as those with an *ICD-10* code referencing sepsis explicitly (eg, R65.2, severe sepsis) following the method defined previously.^23^ Patients were categorized as Asian or Other Pacific Islander, Black, Hispanic, and non-Hispanic White (hereinafter referred to as White) using the Research Triangle Institute race codes.^24^ We excluded patients younger than 65 years (1 372 972), unknown race (103 399), American Indian or Alaska Native (37 974, excluded due to the small sample size), admissions from hospice (879), patients receiving chronic mechanical ventilation (75 201), admissions in December 2021 (76 692), patients in hospitals not in the CMS Impact File (20 866), and subsequent admissions for sepsis within 30 days (271 970) (Figure 1). Admissions in December 2021 were excluded to allow a look-forward time of 30 days for mortality. The final analytic cohort consisted of 5 899 869 observations in 3210 hospitals.

### Statistical Analysis

The primary outcome was a composite of all-cause 30-day mortality (defined as any death within 30 days of admission or beyond 30 days if death occurred during the index admission) and major morbidity (acute myocardial infarction, congestive heart failure, acute kidney failure, stroke, and respiratory failure) occurring after admission (eTable 1 in Supplement 1). The secondary outcome was all-cause 30-day mortality. Major morbidity was identified using *ICD-10* diagnostic codes that were coded as not present at the time of admission, using the present on admission indicator.

We estimated the multivariable logistic regression model

*^f^*[*^E^*(*^Y^_ikt_*)] = β_0_ + β_1_COVIDburden*_kt_* + β_2_RaceEthnic*_ik_* + β_3_COVIDburden*_kt_* × RaceEthnic*_ik_* + β_4_*X_ik_* + β_5_*Z_k_* + β_6_Week*_t_* + β_7_Month*_t_* + β_8_Day*_t_*

where *f* is the logit function, *Y_ikt_* is the composite outcome for patient *i* in hospital *k* admitted at time *t* (week of admission); *COVIDburden_kt_* is a weekly measure of the hospital proportion of Medicare beneficiaries who tested positive for COVID-19 in hospital *k* at time *t*, categorized before the pandemic (January 1, 2016, to January 31, 2020), 0%, 0.1% to 2.0%, 2.1% to 10.0%, 10.1% to 20.0%, 20.1% to 30.0%, 30.1% to 40.0%, and more than 40.0%. We specified the hospital COVID-19 burden as a categorical variable because we assumed a priori that the association between outcomes and the COVID-19 burden would be nonlinear.^18,20,25^ *RaceEthnic_ik_* is a mutually exclusive category for race and ethnicity (Asian, Black, Hispanic, and White). The interaction term between *COVIDburden_kt_* and *RaceEthnic_ik_* is used to examine whether racial and ethnic minoritized individuals experienced greater increases in the rate of the composite outcome as the COVID-19 burden increased compared with White individuals. *X_ik_* is a vector of patient-level covariates (demographic characteristics [age, sex], payer status [Medicare only, dual enrollment in Medicare and Medicaid, Medicare Advantage], admission origin [community, hospital, skilled nursing facility or nursing home, other], frailty [specified separately as malnutrition, senility, cognitive dysfunction, dependent on caregiver], COVID-19, shock, cardiac disease [ST-segment elevation myocardial infarction, non–ST-segment elevation myocardial infarction], congestive heart failure [systolic, diastolic, combined], pulmonary disease [acute respiratory distress syndrome, pulmonary edema, acute respiratory failure], individual Elixhauser comorbidities according to Elixhauser comorbidity index, dialysis, prior procedures). *Z_k_* is a vector of hospital characteristics (number of beds, average daily census, resident-to-bed ratio, disproportionate share hospital [DSH] patient percentage [a higher DSH patient percentage indicates a greater proportion of low-income patients, defined as the sum of the percentages of Medicare inpatient days for patients receiving Supplemental Security Income and inpatient days for patients eligible for Medicaid but not Medicare^26^], availability of extracorporeal membrane oxygenation, and geographic region). *Week_t_* is the underlying weekly time trend, *Month_t_* is a vector of monthly indicators to identify seasonal variation around the weekly time trend, and *Day_t_* is a vector of day-of-the week indicators (eg, Monday) to account for variation due to the day of the week. We then repeated this analysis for the secondary outcome, all-cause 30-day mortality.

We performed additional sensitivity analyses. First, we repeated the analyses after excluding patients with COVID-19. Second, we excluded patients admitted between February and June 2020 to examine changes after the first phase of the pandemic.

Data were analyzed between December 16, 2023, and July 11, 2024. All statistical analyses were performed using Stata, MP version 18.0 (StataCorp LLC). We used a cluster robust variance estimator to account for the clustering of observations within hospitals. We calculated adjusted rates using average marginal effects. The threshold for statistical significance was 2-sided values of *P* < .05.

## Results

### Patient Population

Among the 5 899 869 hospitalizations for sepsis (3 038 612 women [51.5%]; 2 861 257 men [48.5%]; mean [SD] age, 78.2 [8.8] years), there were 177 864 (3.0%) Asian, 664 648 (11.3%) Black, 522 964 (8.9%) Hispanic, and 4 534 393 (76.9%) White individuals (Table). Compared with hospital weekly COVID-19 burden before the pandemic (3 975 502 [67.4%]), 280 748 patients with sepsis (4.8%) were hospitalized during weeks with 0% patients with COVID-19, 243 693 (4.1%) during weeks with 0.1% to 2.0%; 802 722 (13.6%) during weeks with 2.1% to 10.0%; 349 841 (5.9%) during weeks with 10.1% to 20.0%; 151 067 (2.6%) during weeks with 20.1% to 30.0%; 58 260 (1.0%) during weeks with 30.1% to 40.0%, and 38 036 (0.6%) during weeks with more than 40.0%.

**Table.** Patient Characteristics

Characteristic	Patients, No. (%)				
	Total (N=5 899 869)	Asian or Other Pacific Islander (n=177 864)	Black (n=664 648)	Hispanic (n=522 964)	White (n=4 534 393)
Hospital weekly COVID-19 burden					
Before pandemic	3 975 502 (67.4)	118 780 (66.8)	427 091 (64.3)	334 557 (64)	3 095 074 (68.3)
0	280 748 (4.8)	9162 (5.2)	25 169 (3.8)	25 268 (4.8)	221 149 (4.9)
0.1-2.0	243 693 (4.1)	9013 (5.1)	27 644 (4.2)	21 258 (4.1)	185 778 (4.1)
2.1-10.0	802 722 (13.6)	24 323 (13.7)	103 271 (15.5)	74 779 (14.3)	600 349 (13.2)
10.1-20.0	349 841 (5.9)	8972 (5.0)	47 514 (7.2)	34 449 (6.6)	258 906 (5.7)
20.1-30.0	151 067 (2.6)	3955 (2.2)	20 410 (3.1)	16 425 (3.1)	110 277 (2.4)
30.1-40.0	58 260 (1.0)	1841 (1.0)	7486 (1.1)	7937 (1.5)	40 996 (0.9)
>40.0	38 036 (0.6)	1818 (1.0)	6063 (0.9)	8291 (1.6)	21 864 (0.5)
Age, mean (SD), y	78.2 (8.8)	79.5 (9.4)	76.2 (8.9)	77.4 (8.8)	78.6 (8.7)
Sex					
Male	2 861 257 (48.5)	90 557 (50.9)	310 383 (46.7)	256 935 (49.1)	2 203 382 (48.6)
Female	3 038 612 (51.5)	87 307 (49.1)	354 265 (53.3)	266 029 (50.9)	2 331 011 (51.4)
Admission source					
Community	5 183 059 (87.9)	159 989 (90.0)	580 155 (87.3)	477 911 (91.4)	3 965 004 (87.4)
Hospital	287 088 (4.9)	4266 (2.4)	24 901 (3.8)	17 197 (3.3)	240 724 (5.3)
SNF/nursing home	354 147 (6)	11 961 (6.7)	50 869 (7.7)	22 079 (4.2)	269 238 (5.9)
Other	75 575 (1.3)	1648 (0.9)	8723 (1.3)	5777 (1.1)	59 427 (1.3)
Dual eligibility	1 834 746 (31.1)	101 768 (57.2)	345 638 (52.0)	297 928 (57.0)	1 089 412 (24.0)
Medicare Advantage	2 045 857 (34.7)	69 477 (39.1)	270 120 (40.6)	256 000 (49.0)	1 450 260 (32.0)
Functional status/frailty					
Malnutrition	944 138 (16.0)	36 090 (20.3)	148 464 (22.3)	82 525 (15.8)	677 059 (14.9)
Senility	28 096 (0.5)	704 (0.4)	3113 (0.5)	2029 (0.4)	22 250 (0.5)
Cognitive dysfunction	130 036 (2.2)	3366 (1.9)	14 350 (2.2)	9558 (1.8)	102 762 (2.3)
Dependent on caregiver	144 964 (2.5)	6918 (3.9)	33 769 (5.1)	22 985 (4.4)	81 292 (1.8)
COVID-19	273 273 (4.6)	9716 (5.5)	46 383 (7.0)	42 270 (8.1)	174 904 (3.9)
Shock	1 232 188 (20.9)	39 475 (22.2)	153 772 (23.1)	116 220 (22.2)	922 721 (20.4)
Myocardial infarction					
No prior myocardial infarction	5 479 460 (92.9)	163 536 (91.9)	618 184 (93.0)	484 941 (92.7)	4 212 799 (92.9)
Prior ST-segment elevation MI	13 120 (0.2)	513 (0.3)	1484 (0.2)	1288 (0.3)	9835 (0.2)
Prior non–ST-segment elevation MI	209 234 (3.6)	7558 (4.3)	21 858 (3.3)	20 212 (3.9)	159 606 (3.5)
Prior other MI	198 055 (3.4)	6257 (3.5)	23 122 (3.5)	16 523 (3.2)	152 153 (3.4)
Congestive heart failure					
None	4 110 066 (69.7)	133 265 (74.9)	454 590 (68.4)	385 508 (73.7)	3 136 703 (69.2)
Systolic	428 430 (7.3)	9903 (5.6)	53 798 (8.1)	34 241 (6.6)	330 488 (7.3)
Diastolic	791 187 (13.4)	19 963 (11.2)	85 261 (12.8)	54 375 (10.4)	631 588 (13.9)
Systolic and diastolic	189 032 (3.2)	4440 (2.5)	23 688 (3.6)	14 477 (2.8)	146 427 (3.2)
Unspecified	381 154 (6.5)	10 293 (5.8)	47 311 (7.1)	34 363 (6.6)	289 187 (6.4)
Pulmonary					
Acute respiratory distress syndrome	44 515 (0.8)	1986 (1.1)	6648 (1.0)	7515 (1.4)	28 366 (0.6)
Pulmonary edema	62 221 (1.1)	2425 (1.4)	6590 (1.0)	6936 (1.3)	46 270 (1.0)
Pulmonary interstitial disease	118 495 (2.0)	4251 (2.4)	8248 (1.2)	12 862 (2.5)	93 134 (2.1)
Acute respiratory failure	1 381 349 (23.4)	44 755 (25.2)	152 553 (23.0)	124 437 (23.8)	1 059 604 (23.4)
Acute on chronic respiratory failure	484 277 (8.2)	8619 (4.9)	42 278 (6.4)	27 724 (5.3)	405 656 (9.0)
Respiratory failure, unspecified	64 675 (1.1)	2620 (1.5)	8577 (1.3)	7219 (1.4)	46 259 (1.0)
Elixhauser comorbidity index diagnoses					
Alcohol abuse	145 111 (2.5)	1555 (0.9)	18 466 (2.8)	11 557 (2.2)	113 533 (2.5)
Lymphoma	120 281 (2.0)	3461 (2.0)	14 095 (2.1)	10 118 (1.9)	92 607 (2.0)
Leukemia	92 015 (1.6)	2037 (1.2)	7774 (1.2)	5174 (1.0)	77 030 (1.7)
Metastatic cancer	296 354 (5.0)	10 442 (5.9)	39 549 (6.0)	23 476 (4.5)	222 887 (4.9)
Solid tumor	341 964 (5.8)	10 903 (6.1)	37 776 (5.7)	26 345 (5.0)	266 940 (5.9)
Cerebrovascular disease	422 959 (7.2)	17 692 (10.0)	84 374 (12.7)	40 442 (7.7)	280 451 (6.2)
Heart failure	1 919 735 (32.5)	48 193 (27.1)	224 650 (33.8)	146 879 (28.1)	1 500 013 (33.1)
Coagulopathy	812 529 (13.8)	30 344 (17.1)	95 402 (14.4)	79 356 (15.2)	607 427 (13.4)
Dementia	1 300 335 (22)	45 813 (25.8)	185 219 (27.9)	125 723 (24.0)	943 580 (20.8)
Liver disease, mild	265 424 (4.5)	10 859 (6.1)	35 311 (5.3)	31 768 (6.1)	187 486 (4.1)
Liver disease and failure, moderate to severe	102 944 (1.7)	3280 (1.8)	9068 (1.4)	16 085 (3.1)	74 511 (1.6)
Chronic pulmonary disease	1 897 934 (32.2)	42 262 (23.8)	178 770 (26.9)	123 238 (23.6)	1 553 664 (34.3)
Neurologic disorder, other	1 373 620 (23.3)	40 215 (22.6)	190 080 (28.6)	108 550 (20.8)	1 034 775 (22.8)
Seizures and epilepsy	271 123 (4.6)	6491 (3.7)	54 911 (8.3)	24 174 (4.6)	185 547 (4.1)
Paralysis	387 087 (6.6)	17 323 (9.7)	86 828 (13.1)	41 615 (8.0)	241 321 (5.3)
Peripheral vascular disease	599 667 (10.2)	19 586 (11.0)	66 394 (10.0)	52 474 (10.0)	461 213 (10.2)
Pulmonary circulation disease	372 146 (6.3)	9775 (5.5)	45 303 (6.8)	24 150 (4.6)	292 918 (6.5)
Kidney failure, moderate	1 321 214 (22.4)	38 228 (21.5)	158 965 (23.9)	101 289 (19.4)	1 022 732 (22.6)
Kidney failure, severe	539 984 (9.2)	22 260 (12.5)	112 782 (17.0)	68 432 (13.1)	336 510 (7.4)
Peptic ulcer disease with bleeding	91 058 (1.5)	4001 (2.3)	10 221 (1.5)	8432 (1.6)	68 404 (1.5)
Valvular disease	605 352 (10.3)	15 495 (8.7)	47 175 (7.1)	40 098 (7.7)	502 584 (11.1)
Weight loss	997 029 (16.9)	39 044 (22)	157 533 (23.7)	87 801 (16.8)	712 651 (15.7)
Dialysis	173 643 (2.9)	8905 (5.0)	47 774 (7.2)	28 926 (5.5)	88 038 (1.9)
Prior procedures					
PCI	367 598 (6.2)	8759 (4.9)	26 387 (4.0)	24 444 (4.7)	308 008 (6.8)
CABG	366 499 (6.2)	9254 (5.2)	21 332 (3.2)	27 705 (5.3)	308 208 (6.8)
Heart valve surgery	125 271 (2.1)	2085 (1.2)	5775 (0.9)	7166 (1.4)	110 245 (2.4)
Left ventricular assist device	1426 (0.02)	24 (0.01)	212 (0.03)	107 (0.02)	1083 (0.02)
Kidney transplant	26 223 (0.4)	1526 (0.9)	4799 (0.7)	4253 (0.8)	15 645 (0.4)
Heart transplant	3867 (0.1)	99 (0.1)	509 (0.1)	291 (0.1)	2968 (0.1)
Liver transplant	9083 (0.2)	356 (0.2)	613 (0.1)	1317 (0.3)	6797 (0.2)
ECMO availability	1 370 261 (23.2)	42 007 (23.6)	197 944 (29.8)	112 179 (21.5)	1 018 131 (22.5)
No. of beds					
≤50	131 209 (2.2)	1164 (0.7)	7104 (1.1)	6534 (1.3)	116 407 (2.6)
51-149	1 110 048 (18.8)	24 488 (13.8)	78 524 (11.8)	69 550 (13.3)	937 486 (20.7)
150-249	1 356 757 (23.0)	45 421 (25.5)	136 030 (20.5)	120 532 (23.1)	1 054 774 (23.3)
250-499	2 129 976 (36.1)	70 613 (39.7)	253 271 (38.1)	207 299 (39.6)	1 598 793 (35.3)
≥500	1 171 879 (19.9)	36 178 (20.3)	189 719 (28.5)	119 049 (22.8)	826 933 (18.2)
Average daily census					
≤20	177 280 (3.0)	1240 (0.7)	9589 (1.44)	8133 (1.56)	158 318 (3.49)
21-50	527 211 (8.9)	9222 (5.2)	35 594 (5.4)	31 189 (6.0)	451 206 (10.0)
51-150	1 932 163 (32.8)	62 182 (35.0)	181 421 (27.3)	164 536 (31.5)	1 524 024 (33.6)
151-300	1 863 500 (31.6)	60 071 (33.8)	216 143 (32.5)	179 563 (34.3)	1 407 723 (31.1)
≥301	1 399 715 (23.7)	45 149 (25.4)	221 901 (33.4)	139 543 (26.7)	993 122 (21.9)
Resident-to-bed ratio					
0	2 636 251 (44.7)	70 307 (39.5)	232 585 (35)	221 928 (42.4)	2 111 431 (46.6)
>0-0.10	1 540 916 (26.1)	45 867 (25.8)	151 979 (22.9)	150 067 (28.7)	1 193 003 (26.3)
0.11-0.20	593 140 (10.1)	18 437 (10.4)	73 054 (11.0)	42 836 (8.2)	458 813 (10.1)
0.21-0.40	619 211 (10.5)	19 604 (11.0)	95 280 (14.3)	48 405 (9.3)	455 922 (10.1)
>0.40	510 351 (8.7)	23 649 (13.3)	111 750 (16.8)	59 728 (11.4)	315 224 (7.0)
DSH percentage					
0-9.9%	275 125 (4.7)	9602 (5.4)	13 709 (2.1)	19 106 (3.7)	232 708 (5.1)
10.0%-24.9%	2 001 173 (33.9)	44 696 (25.1)	156 168 (23.5)	98 699 (18.9)	1 701 610 (37.5)
25.0%-49.9%	3 075 722 (52.1)	80 059 (45.0)	388 989 (58.5)	239 597 (45.8)	2 367 077 (52.2)
≥50.0%	547 849 (9.3)	43 507 (24.5)	105 782 (15.9)	165 562 (31.7)	232 998 (5.1)
Region					
New England	272 434 (4.6)	4083 (2.3)	11 852 (1.8)	12 127 (2.3)	244 372 (5.4)
Middle Atlantic	809 429 (13.7)	25 237 (14.2)	96 048 (14.5)	63 268 (12.1)	624 876 (13.8)
South Atlantic	1 132 678 (19.2)	14 038 (7.9)	192 585 (29.0)	64 428 (12.3)	861 627 (19.0)
East North Central	782 960 (13.3)	8632 (4.9)	85 529 (12.9)	21 038 (4.0)	667 761 (14.7)
East South Central	545 187 (9.2)	4212 (2.4)	91 267 (13.7)	15 983 (3.1)	433 725 (9.6)
West North Central	380 442 (6.5)	3552 (2.0)	28 295 (4.3)	5335 (1.0)	343 260 (7.6)
West South Central	681 472 (11.6)	11 262 (6.3)	89 660 (13.5)	117 241 (22.4)	463 309 (10.2)
Mountain	363 635 (6.2)	6874 (3.9)	11 994 (1.8)	45 999 (8.8)	298 768 (6.6)
Pacific	913 443 (15.5)	99 972 (56.2)	57 409 (8.6)	159 452 (30.5)	596 610 (13.2)
Puerto Rico	18 189 (0.3)	2 (<0.1)	9 (<0.1)	18 093 (3.46)	85 (<0.1)

Abbreviations: CABG, coronary artery bypass grafting; DSH, disproportionate share hospital; ECMO, extracorporeal membrane oxygenation; MI, myocardial infarction; PCI, percutaneous coronary intervention; SNF, skilled nursing facility.

Asian (101 768 [57.2%]), Black (345 638 [52.0%]), and Hispanic (297 928 [57.0%]) individuals were more likely to be dually enrolled in Medicare and Medicaid than White (1 089 412 [24.0%]) individuals (Table). Asian (9716 [5.5%]), Black (46 383 [7.0%]), and Hispanic (42 270 [8.1%]) individuals were more likely to have COVID-19 compared with White (174 904 [3.9%]) individuals. Asian (8905 [5.0%]), Black (47 774 [7.2%]), and Hispanic (28 926 [5.5%]) individuals were more likely to be receiving chronic dialysis than White (88 038 [1.9%]) individuals. Asian (43 507 [24.5%]), Black (105 782 [15.9%]), and Hispanic (165 562 [31.7%]) individuals were more likely to receive care from a hospital with a DSH percentage greater than 50.0% than White (232 998 [5.1%]) individuals. The hospital characteristics are reported in eTable 2 in Supplement 1.

### Association of Hospital COVID-19 Burden With Death and Major Morbidity

**Figure 2.**
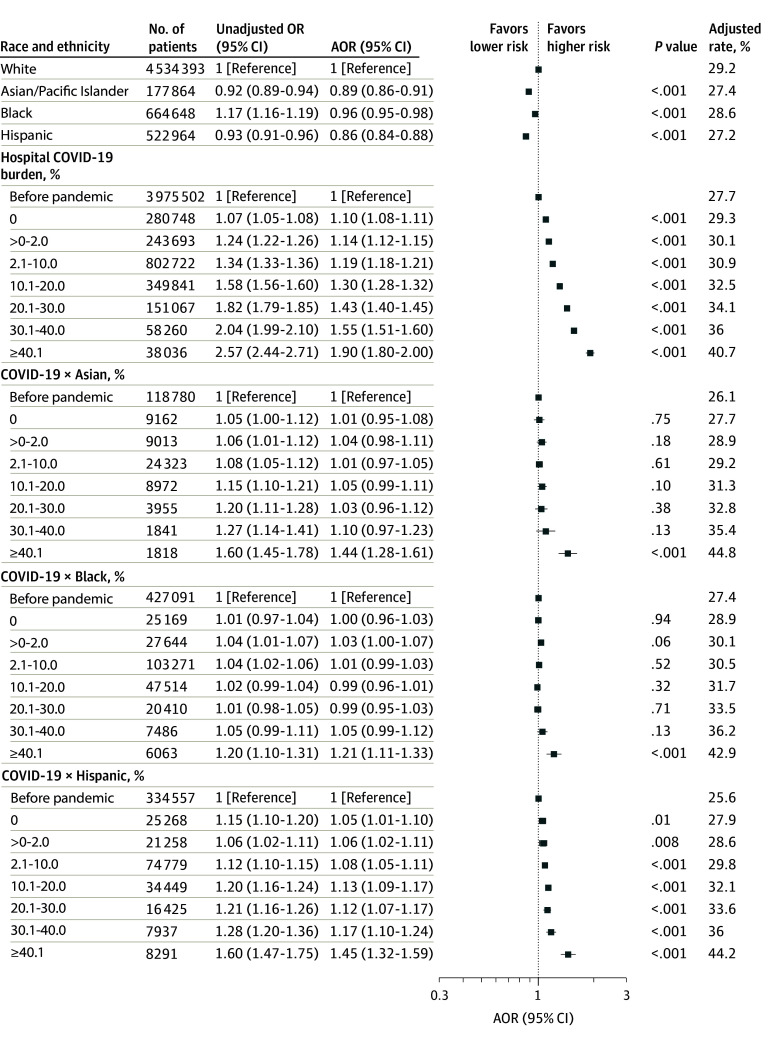
Changes in Mortality or Major Morbidity in Patients With Sepsis as a Function of the Weekly Hospital COVID-19 Burden The model was adjusted for patient demographic characteristics, payer status, site of origin, frailty, COVID-19 diagnosis, dialysis, prior procedures, comorbidities, hospital characteristics, and time trends. The unadjusted model was adjusted for patient demographic characteristics, payer status, site of origin, and time trends. The hospital COVID-19 odds ratios (ORs) quantify the association between the outcome and the hospital COVID-19 burden for White individuals. The interaction term COVID-19 × Black odds ratios quantifies the additional effect observed with the hospital COVID-19 burden for Black individuals compared with White individuals. The other interaction terms are interpreted in a similar fashion. AOR indicates adjusted OR.

Before the pandemic and after adjusting for age, sex, dual enrollment in Medicare and Medicaid, and the source of admission, Black (adjusted odds ratio [AOR], 1.17; 95% CI, 1.16-1.19; *P* < .001) individuals were more likely and Asian (AOR, 0.92; 95% CI, 0.89-0.94; *P* < .001) and Hispanic (AOR, 0.93; 95% CI, 0.91-0.96; *P* < .001) individuals were less likely to experience death or major morbidity than White individuals (Figure 2; eTable 3 in Supplement 1). After adjusting for patient risk and hospital characteristics, Asian (AOR, 0.89; 95% CI, 0.86-0.9; *P* < .001), Black (AOR, 0.96; 95% CI, 0.95-0.98; *P* < .001), and Hispanic (AOR, 0.86; 95% CI, 0.84-0.88; *P* < .001) individuals were less likely to experience death or major morbidity than White individuals (Figure 2; eTable 3 in Supplement 1).

After adjusting for patient risk and hospital characteristics, the risk of death or major morbidity (our composite outcome) for White individuals increased by 43% (AOR, 1.43; 95% CI, 1.40-1.45; *P* < .001) in hospitals with COVID-19 burdens of 20.1% to 30.0%, 55% (AOR, 1.55; 95% CI, 1.51-1.60; *P* < .001) in hospitals with COVID-19 burdens of 30.1% to 40.0%, and 90% (AOR, 1.90; 95% CI, 1.80-2.00; *P* < .001) in hospitals with COVID-19 burdens greater than 40% compared with the prepandemic period (Figure 2; eTable 3 in Supplement 1). These findings were similar when patients with COVID-19 were excluded from the analysis (20.1%-30.0% burden: AOR, 1.41; 95% CI, 1.38-1.44; *P* < .001; 30.1%-40.0% burden: AOR, 1.54; 95% CI, 1.49-1.59; *P* < .001; and >40.0% burden: AOR, 1.81; 95% CI, 1.71-1.91; *P* < .001) (eFigure 1 in Supplement 1).

As the hospital COVID-19 burden increased during the pandemic, Asian, Black, and Hispanic individuals experienced greater increases in mortality and morbidity than White individuals (Figure 2; eTable 3 in Supplement 1). In particular, when the hospital weekly COVID-19 burden was greater than 40.0%, a higher risk of death or morbidity was observed in Asian (44%; AOR, 1.44; 95% CI, 1.28-1.61; *P* < .001), Black (21%; AOR, 1.21; 95% CI, 1.11-1.33; *P* < .001), and Hispanic (45%; AOR, 1.45; 95% CI, 1.32-1.59; *P* < .001) individuals compared with White individuals. Asian individuals also experienced a higher risk of death and morbidity than White individuals when hospital COVID-19 burdens were lower than 40.0% (Figure 2; eTable 3 in Supplement 1). These findings were similar when patients with COVID-19 were excluded from the analysis (eFigure 1 in Supplement 1). However, the magnitude of the effect sizes for risk was reduced when we excluded patients admitted between February and June 2020 (eFigure 3 in Supplement 1).

### Association of Hospital COVID-19 Burden With Disparities in Mortality

**Figure 3.**
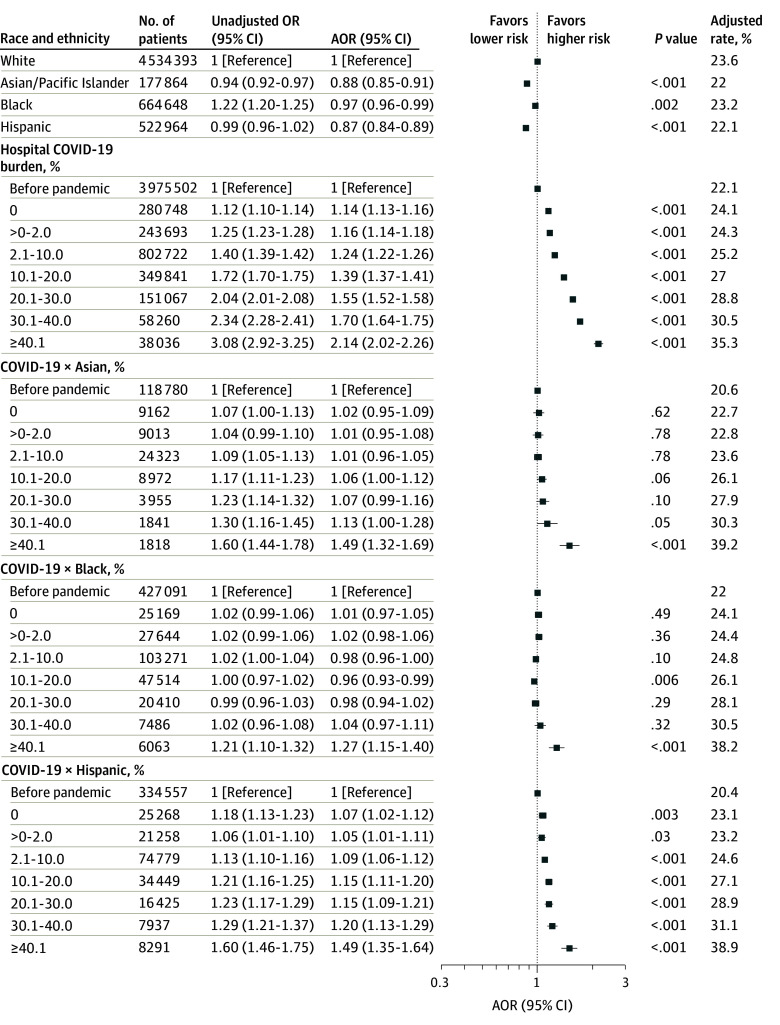
Changes in Mortality in Patients With Sepsis as a Function of the Weekly Hospital COVID-19 Burden The model was adjusted for patient demographic characteristics, payer status, site of origin, frailty, COVID-19 diagnosis, dialysis, prior procedures, comorbidities, hospital characteristics, and time trends. The unadjusted model was adjusted for patient demographic characteristics, payer status, site of origin, and time trends. The hospital COVID-19 odds ratios (ORs) quantify the association between the outcome and the hospital COVID-19 burden for White individuals. The interaction term COVID19 × Black odds ratios quantifies the additional effect observed with the hospital COVID-19 burden for Black individuals compared with White individuals. The other interaction terms are interpreted in a similar fashion. AOR indicates adjusted OR.

In secondary analyses, the risk of death in White patients increased by 55% (AOR, 1.55; 95% CI, 1.52-1.58; *P* < .001) during weeks with COVID-19 burdens of 20.1% to 30.0%, 70% (AOR, 1.70; 95% CI, 1.64-1.75; *P* < .001) during weeks with COVID-19 burdens of 30.1% to 40.0%, and 2.1-fold (AOR, 2.14; 95% CI, 2.02-2.26; *P* < .001) during weeks with COVID-19 burdens greater than 40.0% compared with before the pandemic (Figure 3). These findings were similar when patients with COVID-19 were excluded from the analysis (eFigure 2 in Supplement 1).

Compared with White patients, greater increases in mortality during the pandemic were observed in Asian (49%; AOR, 1.49; 95% CI, 1.32-1.69; *P* < .001), Black (27%; AOR, 1.27; 95% CI, 1.15-1.40; *P* < .001), and Hispanic (49%; AOR, 1.49; 95% CI, 1.35-1.64; *P* < .001) individuals when the hospital weekly COVID-19 burden was greater than 40.0% (Figure 3). These findings were similar when patients with COVID-19 were excluded from the analysis (eFigure 2 in Supplement 1). However, the magnitude of the effect sizes was reduced when we excluded patients admitted between February and June 2020 (eFigure 4 in Supplement 1).

## Discussion

In this cross-sectional study of 5.9 million patients hospitalized with sepsis, patients treated during weeks with an increased inpatient census of patients with COVID-19 experienced higher levels of mortality and major morbidity compared with before the pandemic. We also observed that patients belonging to racial and ethnic minority groups experienced greater increases in adverse outcomes compared with White patients during weeks with a high COVID-19 patient burden. Patients with sepsis treated in hospitals with more than 40% COVID-19 hospital burden experienced rates of mortality and major morbidity that were nearly twice as high as before the pandemic. Asian, Black, and Hispanic individuals experienced mortality and morbidity rates that were 44%, 21%, and 45% higher, respectively, than White individuals during these COVID-19 surges. Findings were similar when limiting our analysis to patients with sepsis who did not have COVID-19. However, the effect sizes found between hospital strain and outcomes and these differences between racial and ethnic minoritized individuals and White individuals were reduced when we excluded patients admitted during the first 5 months of the pandemic. These latter findings may reflect improvements in how hospitals managed the strain of caring for large numbers of patients with COVID-19 over time.

There was a significant increase in adverse clinical outcomes for patients with sepsis during weeks with high COVID-19 burden. Patients with sepsis are among the most critically ill in a hospital and frequently require resource-intensive therapies, such as mechanical ventilation, prone positioning, and renal replacement therapy, and receive higher levels of physician and nurse staffing than patients who are less critically ill. It is thus not surprising that patients with sepsis experienced worse outcomes in crisis and near-crisis situations during the height of the pandemic. What is less evident is why these surges were associated with greater increases in adverse outcomes among patients from racial and ethnic minority populations. One possibility is that resources were inequitably allocated when scarcity emerged. Crisis or near-crisis conditions during the pandemic may have unmasked latent disparities that are only evident in the most critically ill patients because such patients required resources, such as ventilators and trained intensive care unit staff, that may not have been available to all patients who needed them.^18,19,20^

Hospitals operating under crisis standards of care aim to save the most lives. Some patients who would have survived under normal conditions may not survive in crisis or near-crisis situations.^12,27,28^ Under crisis conditions, it is considered necessary, and even acceptable, for hospitals to withhold scarce resources from some patients if other patients are more likely to benefit from these resources.^27^ For example, facilities facing resource shortages during the pandemic implemented contingency levels of care, such as using transport ventilators, boarding critically ill patients in postanesthesia care units, and using nontraditional staff to care for patients.^12^ In providing guidance, the National Academies of Sciences, Engineering, and Medicine acknowledged that “conditions will change as the pandemic spreads nationally, leading to dynamic shifts in standards of care across communities and facilities.”^27^ However, ensuring that difficult decisions related to resource allocation are made with explicit attention to equity is crucial. Whether intentional or not, in the face of such crisis and near-crisis conditions and despite efforts to deliver equitable care,^27^ underlying inequities in the US health care systems may have been exacerbated by the inequitable distribution of scarce resources.

It is also possible that our findings reflect broader inequities in the health care system and differences in the changes in the underlying health status of vulnerable individuals during COVID-19, both of which may be associated with structural and systemic racism across society at large.^29,30,31^ Certainly, the pandemic led to profound racial and ethnic inequities in outcomes; as of March 2023, there were more than 103 million cases and 1.1 million deaths from COVID-19 in the US,^32^ with Black, Hispanic, and other marginalized populations accounting for 36% of COVID-19–associated deaths and 58% of non–COVID-19–associated excess years of life lost.^33,34,35^ Since racial and ethnic minoritized groups disproportionately receive care at hospitals with lower levels of staffing, worse financial reserves, and other financial and organizational challenges, another important area to understand for future work could be the degree to which federal and state programs established to help hospitals during the pandemic appropriately targeted underresourced hospitals that were at exceptionally high risk of strain-related deteriorations in care and clinical outcomes.

Previous studies reported that patients admitted with acute myocardial infarction or stroke or who underwent noncardiac surgery during weeks with a high inpatient census of COVID-19 cases were also more likely to experience worse outcomes compared with those before the pandemic.^18,19,20^ However, in those studies, individuals belonging to racial and ethnic minority groups were not disproportionately affected by the pandemic. Sepsis may differ from these other conditions because of the direct competition for resources needed to treat critically ill patients with COVID-19 or because patients with sepsis have a large burden of social and medical risk factors that make them highly vulnerable to even small changes in care patterns. One study examining 8 teaching hospitals during COVID-19 similarly reported increases in sepsis mortality during the pandemic, although their findings were not stratified by race and ethnicity.^36^ Our study, based on national data, provides evidence that the pandemic was associated with worsening racial and ethnic disparities in the most critically ill patients. These findings may help policymakers and health care leaders formulate specific policies that will promote more equitable health care for critically ill vulnerable individuals.

### Limitations

This study has several limitations. First, it is possible that individuals of racial and ethnic minority populations were more critically ill than White individuals in ways that were not captured using claims-based data. If this were the case, however, Asian, Black, and Hispanic individuals would not have been less likely to die or experience major morbidity before the pandemic in adjusted analyses. Second, in considering baseline disparities in sepsis outcomes, it would have been reasonable to not adjust for disease severity since individuals in racial and ethnic minority groups may present with greater disease burden due to systemic racism. However, since the focus of our study was the interaction of hospital strain and disparities, it was necessary to control for severity to account for potential differences in outcomes due to greater disease severity in racial and ethnic minoritized compared with White individuals. Third, our findings are based on older individuals with Medicare coverage and may not be generalizable to younger individuals or older individuals without Medicare coverage. However, individuals aged 65 years or older account for 65% of sepsis cases despite representing only 12% of the US population,^37^ and 98.9% of adults aged 65 years or older have Medicare coverage.^38^ Fourth, our main findings could have resulted if COVID-19–associated sepsis was more severe in racial and ethnic minoritized individuals than White individuals. However, we found nearly identical findings when we excluded patients with COVID-19 in our sensitivity analyses. Fifth, our study does not identify potential mechanisms for the main findings, and further work is needed to elucidate them. Sixth, we did not include American Indian or Alaska Native people in our study due to sample size limitations. Seventh, the association of outcomes with the weekly hospital COVID-19 burden could be biased upward if more severely ill patients were more likely to be admitted during periods of increased hospital strain. However, it is unlikely that this would differentially affect racial and ethnic minoritized groups. Eighth, our measure of hospital strain may not perfectly capture other measures of resource allocation that directly reflect hospital strain, such as nurse staffing ratios and the use of alternative care settings. As such, our estimates of the differential association of outcomes with hospital strain among individuals of racial and ethnic minorities may be downwardly biased. There is no consensus on the ideal measure of hospital strain: a recent systematic review of 39 studies examining measures of hospital strain during the pandemic revealed 37 different surge metrics.^39^ Ninth, as with other nonrandomized studies, our study cannot be used to make causal inferences.

## Conclusions

In this cross-sectional study of 5.9 million patients with sepsis, patients hospitalized with sepsis during weeks with a greater number of COVID-19 patients were more likely to die or experience major morbidity. Asian, Black, and Hispanic individuals with sepsis experienced greater increases in mortality and morbidity than White individuals as the hospital COVID-19 burden increased. These results highlight the need to reinforce the importance of health equity when health care systems face crisis or near-crisis conditions.

## Notes

Supplement 1. **eFigure 1.** Changes in Mortality or Major Morbidity in Patients With Sepsis as a Function of the Weekly Hospital COVID-19 Burden; This Sensitivity Analysis Excludes Patients With COVID-19 **eFigure 2.** Changes in Mortality in Patients With Sepsis as a Function of the Weekly Hospital COVID-19 Burden; This Sensitivity Analysis Excludes Patients With COVID-19 **eFigure 3.** Changes in Mortality or Major Morbidity in Patients With Sepsis as a Function of the Weekly Hospital COVID-19 Burden; This Sensitivity Analysis Excludes Patients Admitted Between February and June 2020 **eFigure 4.** Changes in Mortality in Patients With Sepsis as a Function of the Weekly Hospital COVID-19 Burden; This Sensitivity Analysis Excludes Patients Admitted Between February and June 2020 **eTable 1.** *ICD-10-CM* Diagnostic Codes **eTable 2.** Hospital Characteristics **eTable 3.** All-Cause 30-Day Mortality and Major Complications

Supplement 2.
Data Sharing Statement
