# Supplementary material for: Hospital Strain During the COVID-19 Pandemic and Outcomes in Older Racial and Ethnic Minority Adults
Source: JAMA Netw Open. 2024 Oct 15;7(10):e2438563. doi: 10.1001/jamanetworkopen.2024.38563 (PMC11581618; doi:10.1001/jamanetworkopen.2024.38563)
Supplement: Supplement 1. — eFigure 1. Changes in Mortality or Major Morbidity in Patients With Sepsis as a Function of the Weekly Hospital COVID-19 Burden; This Sensitivity Analysis Excludes Patients With COVID-19 eFigure 2. Changes in Mortality in Patients With Sepsis as a Function of the Weekly Hospital COVID-19 Burden; This Sensitivity Analysis Excludes Patients With COVID-19 eFigure 3. Changes in Mortality or Major Morbidity in Patients With Sepsis as a Function of the Weekly Hospital COVID-19 Burden; This Sensitivity Analysis Excludes Patients Admitted Between February and June 2020 eFigure 4. Changes in Mortality in Patients With Sepsis as a Function of the Weekly Hospital COVID-19 Burden; This Sensitivity Analysis Excludes Patients Admitted Between February and June 2020 eTable 1. ICD-10-CM Diagnostic Codes eTable 2. Hospital Characteristics eTable 3. All-Cause 30-Day Mortality and Major Complications [file jamanetwopen-e2438563-s001.pdf]

Supplemental Online Content

Glance LG, Joynt Maddox KE, Stone PW, et al. Hospital strain during the COVID-19 pandemic and outcomes in older racial and ethnic minority adults. *JAMA Netw Open.* 2024;7(10):e2438563.
doi:10.1001/jamanetworkopen.2024.38563

eFigure 1. Changes in Mortality or Major Morbidity in Patients With Sepsis as a Function of the Weekly Hospital COVID-19 Burden; This Sensitivity Analysis Excludes Patients With COVID-19

eFigure 2. Changes in Mortality in Patients With Sepsis as a Function of the Weekly Hospital COVID-19 Burden; This Sensitivity Analysis Excludes Patients With COVID-19

eFigure 3. Changes in Mortality or Major Morbidity in Patients With Sepsis as a Function of the Weekly Hospital COVID-19 Burden; This Sensitivity Analysis Excludes Patients Admitted Between February and June 2020

eFigure 4. Changes in Mortality in Patients With Sepsis as a Function of the Weekly Hospital COVID-19 Burden; This Sensitivity Analysis Excludes Patients Admitted Between February and June 2020

eTable 1. *ICD-10-CM* Diagnostic Codes

eTable 2. Hospital Characteristics

eTable 3. All-Cause 30-Day Mortality and Major Complications

This supplemental material has been provided by the authors to give readers additional information about their work.

© 2024 Glance LG et al. *JAMA Network Open*.

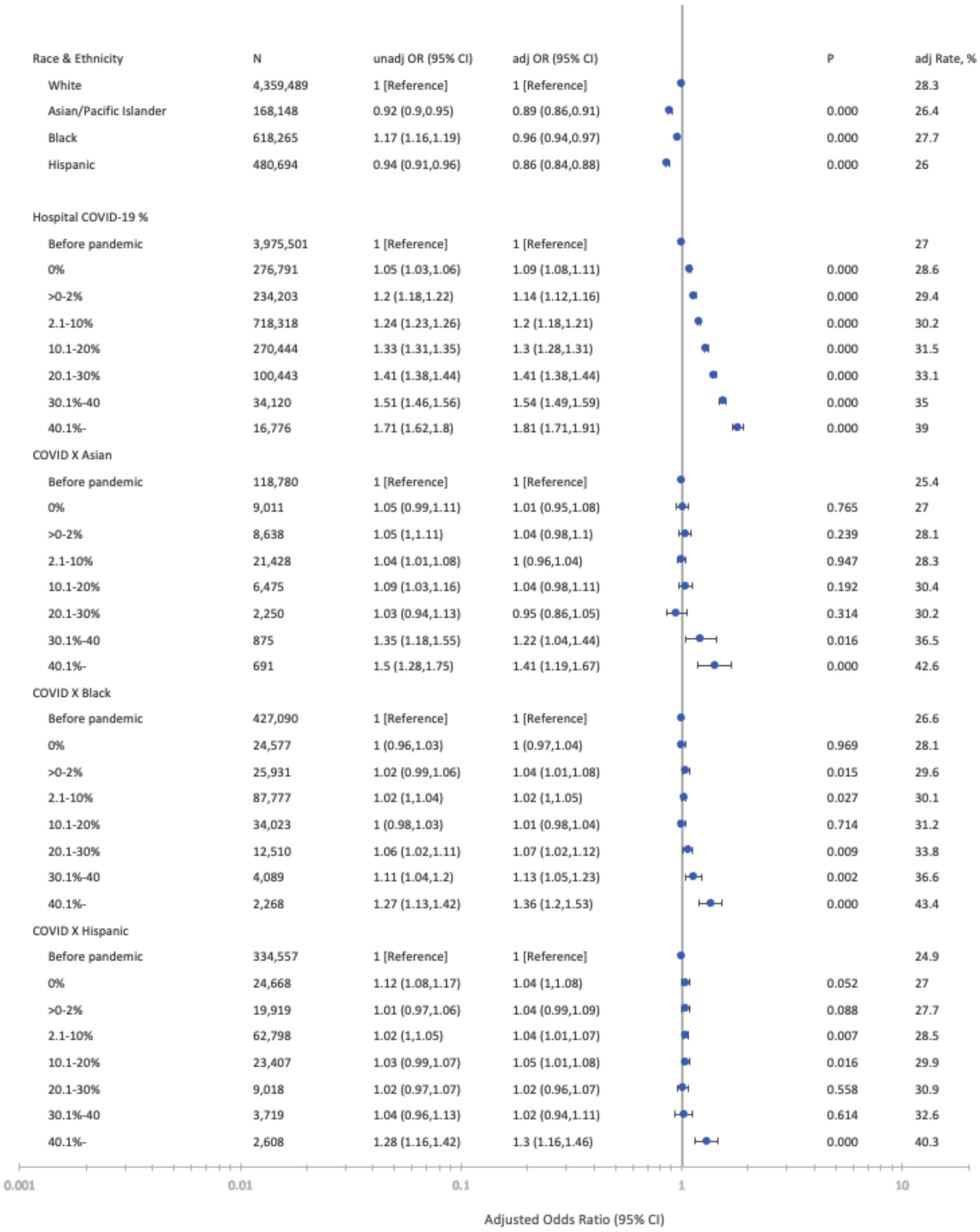

Race & Ethnicity	N	unadj OR (95% CI)	adj OR (95% CI)	P	adj Rate, %
White	4,359,489	1 [Reference]	1 [Reference]		28.3
Asian/Pacific Islander	168,148	0.92 (0.9,0.95)	0.89 (0.86,0.91)	0.000	26.4
Black	618,265	1.17 (1.16,1.19)	0.96 (0.94,0.97)	0.000	27.7
Hispanic	480,694	0.94 (0.91,0.96)	0.86 (0.84,0.88)	0.000	26
Hospital COVID-19 %					
Before pandemic	3,975,501	1 [Reference]	1 [Reference]		27
0%	276,791	1.05 (1.03,1.06)	1.09 (1.08,1.11)	0.000	28.6
>0-2%	234,203	1.2 (1.18,1.22)	1.14 (1.12,1.16)	0.000	29.4
2.1-10%	718,318	1.24 (1.23,1.26)	1.2 (1.18,1.21)	0.000	30.2
10.1-20%	270,444	1.33 (1.31,1.35)	1.3 (1.28,1.31)	0.000	31.5
20.1-30%	100,443	1.41 (1.38,1.44)	1.41 (1.38,1.44)	0.000	33.1
30.1%-40	34,120	1.51 (1.46,1.56)	1.54 (1.49,1.59)	0.000	35
40.1%-	16,776	1.71 (1.62,1.8)	1.81 (1.71,1.91)	0.000	39
COVID X Asian					
Before pandemic	118,780	1 [Reference]	1 [Reference]		25.4
0%	9,011	1.05 (0.99,1.11)	1.01 (0.95,1.08)	0.765	27
>0-2%	8,638	1.05 (1,1.11)	1.04 (0.98,1.1)	0.239	28.1
2.1-10%	21,428	1.04 (1.01,1.08)	1 (0.96,1.04)	0.947	28.3
10.1-20%	6,475	1.09 (1.03,1.16)	1.04 (0.98,1.11)	0.192	30.4
20.1-30%	2,250	1.03 (0.94,1.13)	0.95 (0.86,1.05)	0.314	30.2
30.1%-40	875	1.35 (1.18,1.55)	1.22 (1.04,1.44)	0.016	36.5
40.1%-	691	1.5 (1.28,1.75)	1.41 (1.19,1.67)	0.000	42.6
COVID X Black					
Before pandemic	427,090	1 [Reference]	1 [Reference]		26.6
0%	24,577	1 (0.96,1.03)	1 (0.97,1.04)	0.969	28.1
>0-2%	25,931	1.02 (0.99,1.06)	1.04 (1.01,1.08)	0.015	29.6
2.1-10%	87,777	1.02 (1,1.04)	1.02 (1,1.05)	0.027	30.1
10.1-20%	34,023	1 (0.98,1.03)	1.01 (0.98,1.04)	0.714	31.2
20.1-30%	12,510	1.06 (1.02,1.11)	1.07 (1.02,1.12)	0.009	33.8
30.1%-40	4,089	1.11 (1.04,1.2)	1.13 (1.05,1.23)	0.002	36.6
40.1%-	2,268	1.27 (1.13,1.42)	1.36 (1.2,1.53)	0.000	43.4
COVID X Hispanic					
Before pandemic	334,557	1 [Reference]	1 [Reference]		24.9
0%	24,668	1.12 (1.08,1.17)	1.04 (1,1.08)	0.052	27
>0-2%	19,919	1.01 (0.97,1.06)	1.04 (0.99,1.09)	0.088	27.7
2.1-10%	62,798	1.02 (1,1.05)	1.04 (1.01,1.07)	0.007	28.5
10.1-20%	23,407	1.03 (0.99,1.07)	1.05 (1.01,1.08)	0.016	29.9
20.1-30%	9,018	1.02 (0.97,1.07)	1.02 (0.96,1.07)	0.558	30.9
30.1%-40	3,719	1.04 (0.96,1.13)	1.02 (0.94,1.11)	0.614	32.6
40.1%-	2,608	1.28 (1.16,1.42)	1.3 (1.16,1.46)	0.000	40.3

Supplement Figure 1. Changes in Mortality or Major Morbidity in Patients With Sepsis as a Function of the Weekly Hospital COVID-19 Burden. This sensitivity analysis excludes patients with COVID-19.

The model was adjusted for patient demographics, payer status, site of origin, frailty, COVID-19, dialysis, prior procedures, comorbidities, hospital characteristics, and time trends. The *unadjusted model* was adjusted for patient demographics, payer status, site of origin, and time trends.

© 2024 Glance LG et al. *JAMA Network Open*.

The *Hospital COVID-19 %* odds ratios quantifies the association between the outcome and the hospital COVID-19 burden for White individuals. The interaction term *COVID x Black* odd ratios quantifies the additional effect of the hospital COVID-19 burden for Black individuals compared to White individuals. The other interaction terms are interpreted in a similar fashion.

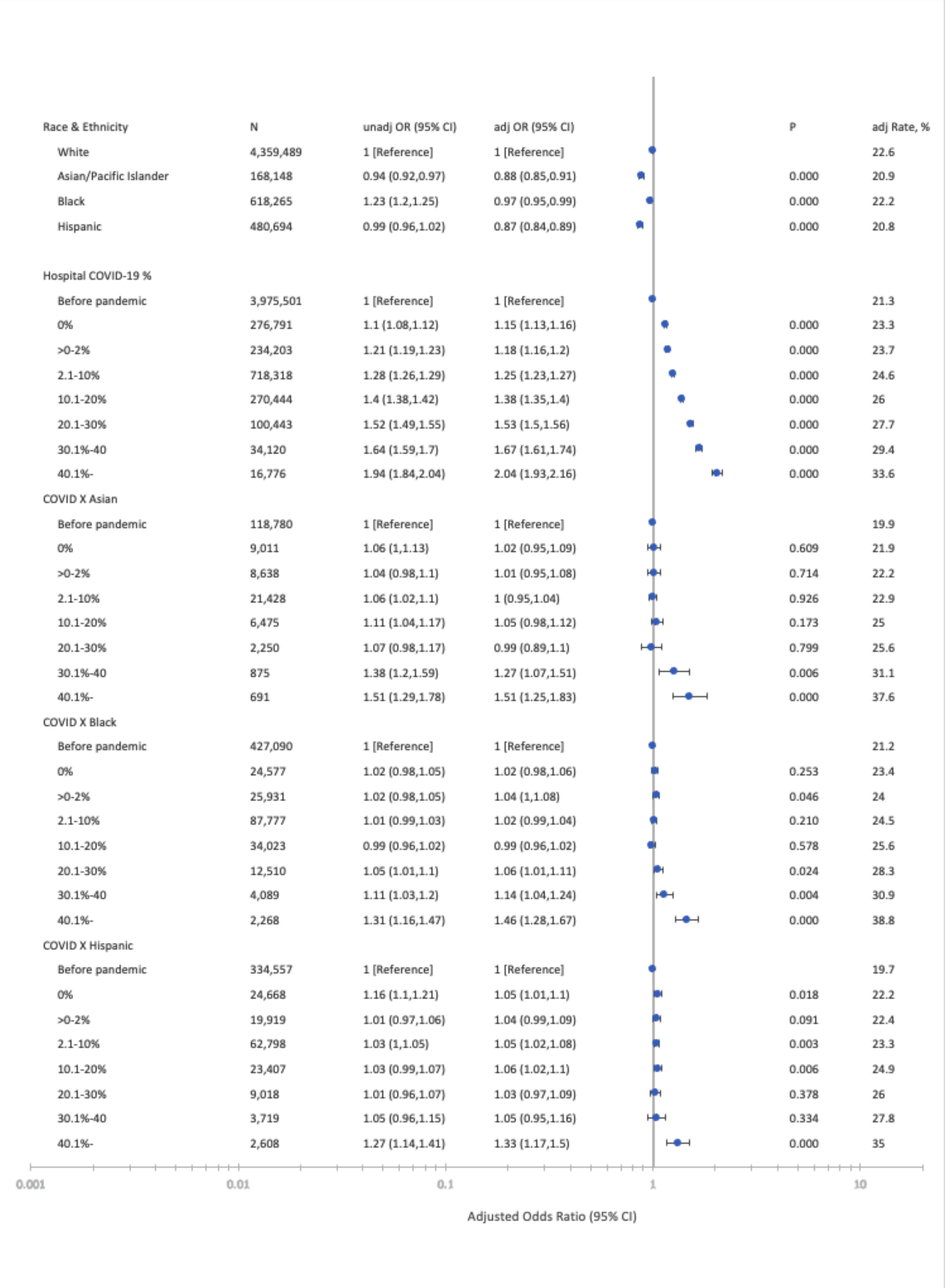

Race & Ethnicity	N	unadj OR (95% CI)	adj OR (95% CI)	P	adj Rate, %
White	4,359,489	1 [Reference]	1 [Reference]		22.6
Asian/Pacific Islander	168,148	0.94 (0.92,0.97)	0.88 (0.85,0.91)	0.000	20.9
Black	618,265	1.23 (1.2,1.25)	0.97 (0.95,0.99)	0.000	22.2
Hispanic	480,694	0.99 (0.96,1.02)	0.87 (0.84,0.89)	0.000	20.8
Hospital COVID-19 %					
Before pandemic	3,975,501	1 [Reference]	1 [Reference]		21.3
0%	276,791	1.1 (1.08,1.12)	1.15 (1.13,1.16)	0.000	23.3
>0-2%	234,203	1.21 (1.19,1.23)	1.18 (1.16,1.2)	0.000	23.7
2.1-10%	718,318	1.28 (1.26,1.29)	1.25 (1.23,1.27)	0.000	24.6
10.1-20%	270,444	1.4 (1.38,1.42)	1.38 (1.35,1.4)	0.000	26
20.1-30%	100,443	1.52 (1.49,1.55)	1.53 (1.5,1.56)	0.000	27.7
30.1%-40	34,120	1.64 (1.59,1.7)	1.67 (1.61,1.74)	0.000	29.4
40.1%-	16,776	1.94 (1.84,2.04)	2.04 (1.93,2.16)	0.000	33.6
COVID X Asian					
Before pandemic	118,780	1 [Reference]	1 [Reference]		19.9
0%	9,011	1.06 (1,1.13)	1.02 (0.95,1.09)	0.609	21.9
>0-2%	8,638	1.04 (0.98,1.1)	1.01 (0.95,1.08)	0.714	22.2
2.1-10%	21,428	1.06 (1.02,1.1)	1 (0.95,1.04)	0.926	22.9
10.1-20%	6,475	1.11 (1.04,1.17)	1.05 (0.98,1.12)	0.173	25
20.1-30%	2,250	1.07 (0.98,1.17)	0.99 (0.89,1.1)	0.799	25.6
30.1%-40	875	1.38 (1.2,1.59)	1.27 (1.07,1.51)	0.006	31.1
40.1%-	691	1.51 (1.29,1.78)	1.51 (1.25,1.83)	0.000	37.6
COVID X Black					
Before pandemic	427,090	1 [Reference]	1 [Reference]		21.2
0%	24,577	1.02 (0.98,1.05)	1.02 (0.98,1.06)	0.253	23.4
>0-2%	25,931	1.02 (0.98,1.05)	1.04 (1,1.08)	0.046	24
2.1-10%	87,777	1.01 (0.99,1.03)	1.02 (0.99,1.04)	0.210	24.5
10.1-20%	34,023	0.99 (0.96,1.02)	0.99 (0.96,1.02)	0.578	25.6
20.1-30%	12,510	1.05 (1.01,1.1)	1.06 (1.01,1.11)	0.024	28.3
30.1%-40	4,089	1.11 (1.03,1.2)	1.14 (1.04,1.24)	0.004	30.9
40.1%-	2,268	1.31 (1.16,1.47)	1.46 (1.28,1.67)	0.000	38.8
COVID X Hispanic					
Before pandemic	334,557	1 [Reference]	1 [Reference]		19.7
0%	24,668	1.16 (1.1,1.21)	1.05 (1.01,1.1)	0.018	22.2
>0-2%	19,919	1.01 (0.97,1.06)	1.04 (0.99,1.09)	0.091	22.4
2.1-10%	62,798	1.03 (1,1.05)	1.05 (1.02,1.08)	0.003	23.3
10.1-20%	23,407	1.03 (0.99,1.07)	1.06 (1.02,1.1)	0.006	24.9
20.1-30%	9,018	1.01 (0.96,1.07)	1.03 (0.97,1.09)	0.378	26
30.1%-40	3,719	1.05 (0.96,1.15)	1.05 (0.95,1.16)	0.334	27.8
40.1%-	2,608	1.27 (1.14,1.41)	1.33 (1.17,1.5)	0.000	35

Supplement Figure 2. Changes in Mortality in Patients With Sepsis as a Function of the Weekly Hospital COVID-19 Burden. This sensitivity analysis excludes patients with COVID-19.

© 2024 Glance LG et al. *JAMA Network Open*.

The model was adjusted for patient demographics, payer status, site of origin, frailty, COVID-19, dialysis, prior procedures, comorbidities, hospital characteristics, and time trends. The *unadjusted model* was adjusted for patient demographics, payer status, site of origin, and time trends.

The *Hospital COVID-19 %* odds ratios quantifies the association between the outcome and the hospital COVID-19 burden for White individuals. The interaction term *COVID x Black* odd ratios quantifies the additional effect of the hospital COVID-19 burden for Black individuals compared to White individuals. The other interaction terms are interpreted in a similar fashion.

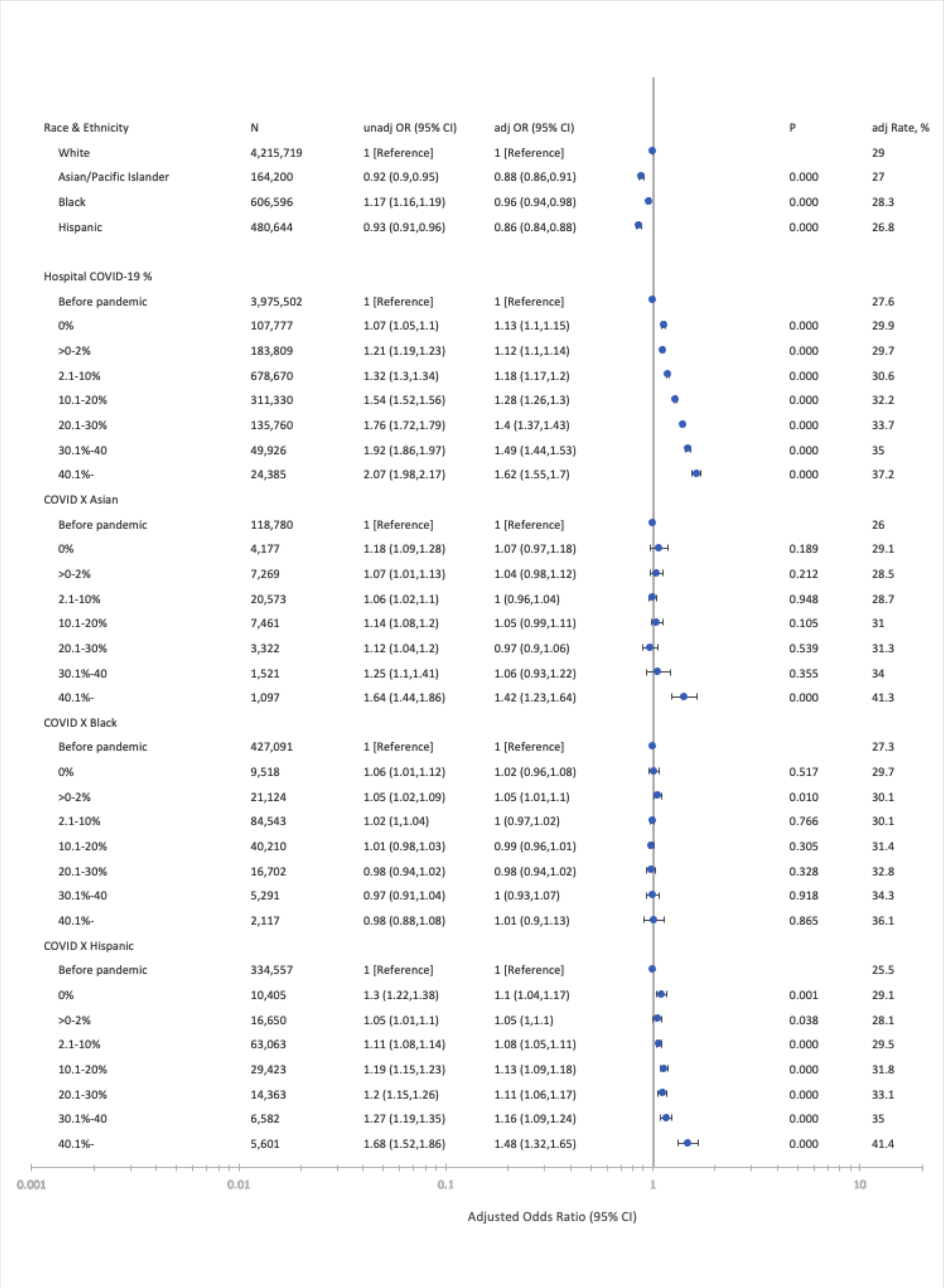

Race & Ethnicity	N	unadj OR (95% CI)	adj OR (95% CI)	P	adj Rate, %
White	4,215,719	1 [Reference]	1 [Reference]		29
Asian/Pacific Islander	164,200	0.92 (0.9,0.95)	0.88 (0.86,0.91)	0.000	27
Black	606,596	1.17 (1.16,1.19)	0.96 (0.94,0.98)	0.000	28.3
Hispanic	480,644	0.93 (0.91,0.96)	0.86 (0.84,0.88)	0.000	26.8
Hospital COVID-19 %					
Before pandemic	3,975,502	1 [Reference]	1 [Reference]		27.6
0%	107,777	1.07 (1.05,1.1)	1.13 (1.1,1.15)	0.000	29.9
>0-2%	183,809	1.21 (1.19,1.23)	1.12 (1.1,1.14)	0.000	29.7
2.1-10%	678,670	1.32 (1.3,1.34)	1.18 (1.17,1.2)	0.000	30.6
10.1-20%	311,330	1.54 (1.52,1.56)	1.28 (1.26,1.3)	0.000	32.2
20.1-30%	135,760	1.76 (1.72,1.79)	1.4 (1.37,1.43)	0.000	33.7
30.1%-40	49,926	1.92 (1.86,1.97)	1.49 (1.44,1.53)	0.000	35
40.1%-	24,385	2.07 (1.98,2.17)	1.62 (1.55,1.7)	0.000	37.2
COVID X Asian					
Before pandemic	118,780	1 [Reference]	1 [Reference]		26
0%	4,177	1.18 (1.09,1.28)	1.07 (0.97,1.18)	0.189	29.1
>0-2%	7,269	1.07 (1.01,1.13)	1.04 (0.98,1.12)	0.212	28.5
2.1-10%	20,573	1.06 (1.02,1.1)	1 (0.96,1.04)	0.948	28.7
10.1-20%	7,461	1.14 (1.08,1.2)	1.05 (0.99,1.11)	0.105	31
20.1-30%	3,322	1.12 (1.04,1.2)	0.97 (0.9,1.06)	0.539	31.3
30.1%-40	1,521	1.25 (1.1,1.41)	1.06 (0.93,1.22)	0.355	34
40.1%-	1,097	1.64 (1.44,1.86)	1.42 (1.23,1.64)	0.000	41.3
COVID X Black					
Before pandemic	427,091	1 [Reference]	1 [Reference]		27.3
0%	9,518	1.06 (1.01,1.12)	1.02 (0.96,1.08)	0.517	29.7
>0-2%	21,124	1.05 (1.02,1.09)	1.05 (1.01,1.1)	0.010	30.1
2.1-10%	84,543	1.02 (1,1.04)	1 (0.97,1.02)	0.766	30.1
10.1-20%	40,210	1.01 (0.98,1.03)	0.99 (0.96,1.01)	0.305	31.4
20.1-30%	16,702	0.98 (0.94,1.02)	0.98 (0.94,1.02)	0.328	32.8
30.1%-40	5,291	0.97 (0.91,1.04)	1 (0.93,1.07)	0.918	34.3
40.1%-	2,117	0.98 (0.88,1.08)	1.01 (0.9,1.13)	0.865	36.1
COVID X Hispanic					
Before pandemic	334,557	1 [Reference]	1 [Reference]		25.5
0%	10,405	1.3 (1.22,1.38)	1.1 (1.04,1.17)	0.001	29.1
>0-2%	16,650	1.05 (1.01,1.1)	1.05 (1,1.1)	0.038	28.1
2.1-10%	63,063	1.11 (1.08,1.14)	1.08 (1.05,1.11)	0.000	29.5
10.1-20%	29,423	1.19 (1.15,1.23)	1.13 (1.09,1.18)	0.000	31.8
20.1-30%	14,363	1.2 (1.15,1.26)	1.11 (1.06,1.17)	0.000	33.1
30.1%-40	6,582	1.27 (1.19,1.35)	1.16 (1.09,1.24)	0.000	35
40.1%-	5,601	1.68 (1.52,1.86)	1.48 (1.32,1.65)	0.000	41.4

© 2024 Glance LG et al. *JAMA Network Open*.

Supplement Figure 3. Changes in Mortality or Major Morbidity in Patients With Sepsis As a Function of the Weekly Hospital COVID-19 Burden. This sensitivity analysis excludes patients admitted between February and June, 2020.

The model was adjusted for patient demographics, payer status, site of origin, frailty, COVID-19, dialysis, prior procedures, comorbidities, hospital characteristics, and time trends. The *unadjusted model* was adjusted for patient demographics, payer status, site of origin, and time trends.

The *Hospital COVID-19 %* odds ratios quantifies the association between the outcome and the hospital COVID-19 burden for White individuals. The interaction term *COVID x Black* odd ratios quantifies the additional effect of the hospital COVID-19 burden for Black individuals compared to White individuals. The other interaction terms are interpreted in a similar fashion.

© 2024 Glance LG et al. *JAMA Network Open*.

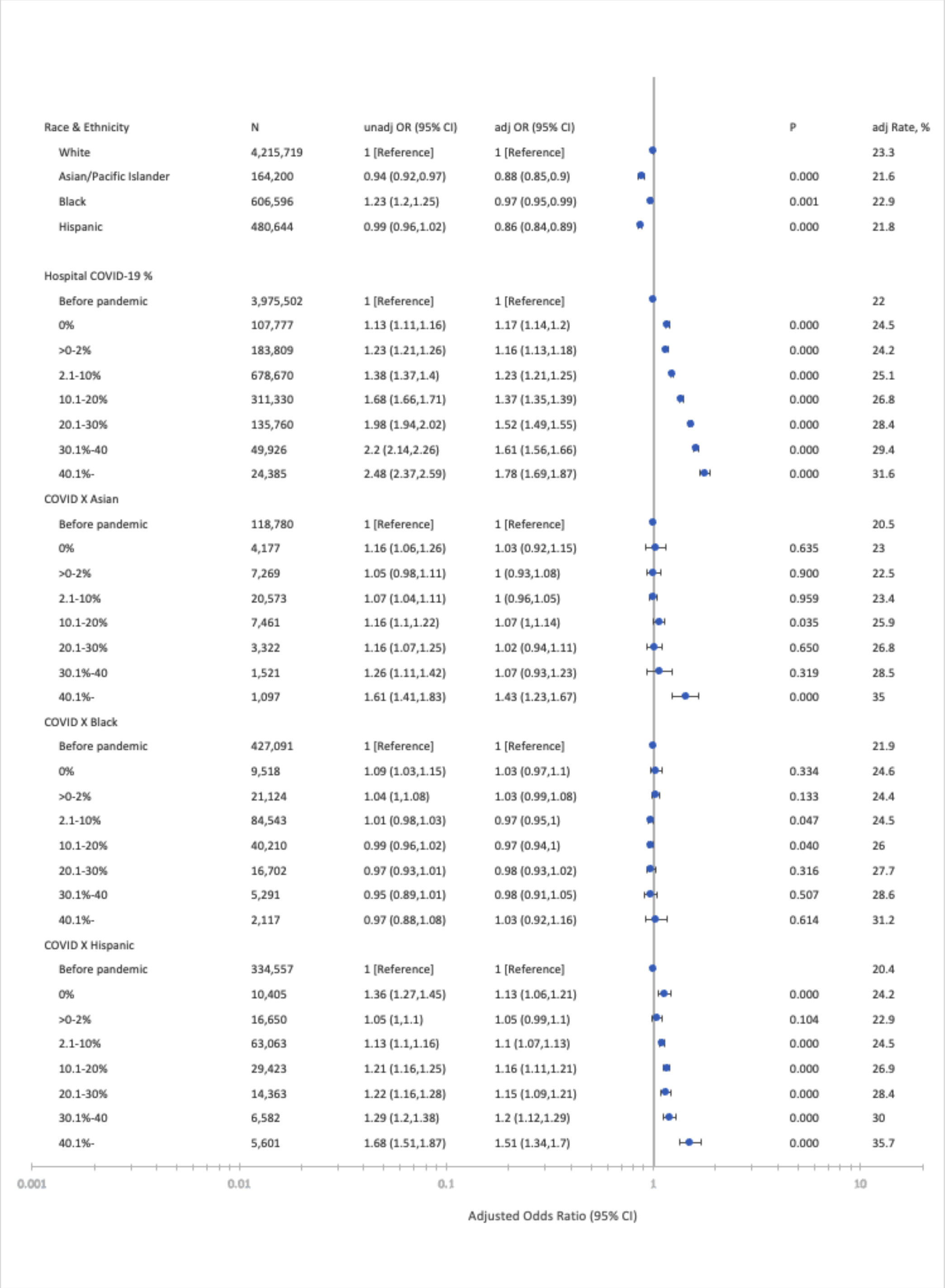

Race & Ethnicity	N	unadj OR (95% CI)	adj OR (95% CI)	P	adj Rate, %
White	4,215,719	1 [Reference]	1 [Reference]		23.3
Asian/Pacific Islander	164,200	0.94 (0.92,0.97)	0.88 (0.85,0.9)	0.000	21.6
Black	606,596	1.23 (1.2,1.25)	0.97 (0.95,0.99)	0.001	22.9
Hispanic	480,644	0.99 (0.96,1.02)	0.86 (0.84,0.89)	0.000	21.8
Hospital COVID-19 %					
Before pandemic	3,975,502	1 [Reference]	1 [Reference]		22
0%	107,777	1.13 (1.11,1.16)	1.17 (1.14,1.2)	0.000	24.5
>0-2%	183,809	1.23 (1.21,1.26)	1.16 (1.13,1.18)	0.000	24.2
2.1-10%	678,670	1.38 (1.37,1.4)	1.23 (1.21,1.25)	0.000	25.1
10.1-20%	311,330	1.68 (1.66,1.71)	1.37 (1.35,1.39)	0.000	26.8
20.1-30%	135,760	1.98 (1.94,2.02)	1.52 (1.49,1.55)	0.000	28.4
30.1%-40	49,926	2.2 (2.14,2.26)	1.61 (1.56,1.66)	0.000	29.4
40.1%-	24,385	2.48 (2.37,2.59)	1.78 (1.69,1.87)	0.000	31.6
COVID X Asian					
Before pandemic	118,780	1 [Reference]	1 [Reference]		20.5
0%	4,177	1.16 (1.06,1.26)	1.03 (0.92,1.15)	0.635	23
>0-2%	7,269	1.05 (0.98,1.11)	1 (0.93,1.08)	0.900	22.5
2.1-10%	20,573	1.07 (1.04,1.11)	1 (0.96,1.05)	0.959	23.4
10.1-20%	7,461	1.16 (1.1,1.22)	1.07 (1,1.14)	0.035	25.9
20.1-30%	3,322	1.16 (1.07,1.25)	1.02 (0.94,1.11)	0.650	26.8
30.1%-40	1,521	1.26 (1.11,1.42)	1.07 (0.93,1.23)	0.319	28.5
40.1%-	1,097	1.61 (1.41,1.83)	1.43 (1.23,1.67)	0.000	35
COVID X Black					
Before pandemic	427,091	1 [Reference]	1 [Reference]		21.9
0%	9,518	1.09 (1.03,1.15)	1.03 (0.97,1.1)	0.334	24.6
>0-2%	21,124	1.04 (1,1.08)	1.03 (0.99,1.08)	0.133	24.4
2.1-10%	84,543	1.01 (0.98,1.03)	0.97 (0.95,1)	0.047	24.5
10.1-20%	40,210	0.99 (0.96,1.02)	0.97 (0.94,1)	0.040	26
20.1-30%	16,702	0.97 (0.93,1.01)	0.98 (0.93,1.02)	0.316	27.7
30.1%-40	5,291	0.95 (0.89,1.01)	0.98 (0.91,1.05)	0.507	28.6
40.1%-	2,117	0.97 (0.88,1.08)	1.03 (0.92,1.16)	0.614	31.2
COVID X Hispanic					
Before pandemic	334,557	1 [Reference]	1 [Reference]		20.4
0%	10,405	1.36 (1.27,1.45)	1.13 (1.06,1.21)	0.000	24.2
>0-2%	16,650	1.05 (1,1.1)	1.05 (0.99,1.1)	0.104	22.9
2.1-10%	63,063	1.13 (1.1,1.16)	1.1 (1.07,1.13)	0.000	24.5
10.1-20%	29,423	1.21 (1.16,1.25)	1.16 (1.11,1.21)	0.000	26.9
20.1-30%	14,363	1.22 (1.16,1.28)	1.15 (1.09,1.21)	0.000	28.4
30.1%-40	6,582	1.29 (1.2,1.38)	1.2 (1.12,1.29)	0.000	30
40.1%-	5,601	1.68 (1.51,1.87)	1.51 (1.34,1.7)	0.000	35.7

Supplement Figure 4. Changes in Mortality in Patients With Sepsis As a Function of the Weekly Hospital COVID-19 Burden. This sensitivity analysis excludes patients admitted between February and June, 2020.

The model was adjusted for patient demographics, payer status, site of origin, frailty, COVID-19, dialysis, prior procedures, comorbidities, hospital characteristics, and time trends. The *unadjusted model* was adjusted for patient demographics, payer status, site of origin, and time trends.

© 2024 Glance LG et al. *JAMA Network Open*.

The *Hospital COVID-19 %* odds ratios quantifies the association between the outcome and the hospital COVID-19 burden for White individuals. The interaction term *COVID x Black* odd ratios quantifies the additional effect of the hospital COVID-19 burden for Black individuals compared to White individuals. The other interaction terms are interpreted in a similar fashion.

© 2024 Glance LG et al. *JAMA Network Open*.

**Supplement Table 1.** ICD-10-CM codes used to identifiy patient characteristics.	
Sepsis	
	A02.1; A20.7; A21.7;A22.7; A24.1; A26.7; A28.2; A32.7; A39.4; A40; A41; A42.7; A54.86; B00.7; B37.7; R65,2
Compllcations	
Acute myocardial infarction	I21; I22
Congestive heart failure	I50
Acute renal faiure	N17
Stroke	I60; I61; I62; I63; I69
Respiratory failure	J96.0; J96.2; J96.9
Frailty	
Wheel-chair	Z99.3
Supplemental oxygen	Z99.81
Dependent on provider	Z74
History of falls	W00, W01,W02,W04,W05,W06, W07,W08,W09,W10, W11,W12,W13,W14,W15,W16,W17,W18,W19
Fecal incontinence	R15
Pre-existing Conditions	
Shock	
Myocardial infarction	
Prior ST-segment elevation MI	I210; I220; I211; I221; I212; I228; I213; I229
Prior Non-ST-segment elevation MI	I214; I222
Prior other MI	I219; I21A
Congestive heart failure	
Systolic CHF	I502
Diastolic CHF	I503
Systolic and diastolic CHF	I504
Unspecified CHF	I501; I509; I5089
Pulmonary	
Acute respiratory distress syndrome	J80
Pulmonary edema	J81
Pulmonary interstitial disease	J84
Acute respiratory failure	J960
Acute on chronic respiratory failure	J962
Respiratory failure, unspecified	J969
COVID-19	U071
Prior procedures	
PCI	Z9861; Z955
CABG	Z951
Heart valve surgery	Z952;Z953;Z954
Left ventricular asist device	Z95811
Kidney transplant	Z940
Heart transplant	Z941
Liver transplant	Z944

© 2024 Glance LG et al. *JAMA Network Open*.

Supplement Table 2. Hospital Characteristics.

Characteristics	Hospitals, No. (%)
Total number of beds	
<50	547 (17)
51-149	1,140 (35.5)
150-249	651 (20.3)
250-499	651 (20.3)
500-	221 (6.9)
Average daily census	
<20	719 (22.4)
21-50	604 (18.8)
51-150	1,046 (32.6)
151-300	555 (17.3)
301-	286 (8.9)
Disproportionate share percentage (DSH)	
0-9.9%	250 (7.8)
10.0-24.9%	1,098 (34.2)
25.0-49.9%	1,517 (47.3)
50.0%-	345 (10.8)
Resident-to-bed ratio	
0	2,103 (65.5)
>0-0.10	519 (16.2)
0.11-0.20	203 (6.3)
0.21-0.40	202 (6.3)
0.41-	183 (5.7)
ECMO availability	323 (10.1)
Region	
New England	132 (4.1)
Middle Atlantic	352 (11)
South Atlantic	534 (16.6)
East North Central	437 (13.6)
East South Central	350 (10.9)
West North Central	256 (8)
West South Central	486 (15.1)
Mountain	222 (6.9)
Pacific	391 (12.2)
Puerto Rico	50 (1.6)

© 2024 Glance LG et al. *JAMA Network Open*.

Supplement Table 3. 30-day Mortality and Morbidity

	Unadusted		Patient Charactistics		Patient and Hospital Characteristics	
	OR (95% CI)	P	OR (95% CI)	P	OR (95% CI)	P
Race						
White	reference		reference		reference	
Asian/Pacific Islander	0.92 (0.89 ,0.94)	0.000	0.81 (0.78 ,0.83)	0.000	0.89 (0.86 ,0.91)	0.000
Black	1.17 (1.16 ,1.19)	0.000	1 (0.98 ,1.01)	0.689	0.96 (0.95 ,0.98)	0.000
Hispanic	0.93 (0.91 ,0.96)	0.000	0.87 (0.85 ,0.9)	0.000	0.86 (0.84 ,0.88)	0.000
Hospital weekly COVID-19 burden (%)						
Before pandemic	reference		reference		reference	
0%	1.07 (1.05 ,1.08)	0.000	1.08 (1.06 ,1.09)	0.000	1.1 (1.08 ,1.11)	0.000
0.1-2.0%	1.24 (1.22 ,1.26)	0.000	1.13 (1.11 ,1.15)	0.000	1.14 (1.12 ,1.15)	0.000
2.1-10.0%	1.34 (1.33 ,1.36)	0.000	1.2 (1.18 ,1.21)	0.000	1.19 (1.18 ,1.21)	0.000
10.1-20.0%	1.58 (1.56 ,1.6)	0.000	1.31 (1.29 ,1.33)	0.000	1.3 (1.28 ,1.32)	0.000
20.1-30.0%	1.82 (1.79 ,1.85)	0.000	1.44 (1.41 ,1.46)	0.000	1.43 (1.4 ,1.45)	0.000
30.1-40.0%	2.04 (1.99 ,2.1)	0.000	1.55 (1.51 ,1.6)	0.000	1.55 (1.51 ,1.6)	0.000
40.1%-	2.57 (2.44 ,2.71)	0.000	1.86 (1.76 ,1.97)	0.000	1.9 (1.8 ,2)	0.000
Hospital COVID-19 X Asian/Pacific Islander						
Before pandemic	reference		reference		reference	
0%	1.05 (1 ,1.12)	0.073	1 (0.94 ,1.06)	0.935	1.01 (0.95 ,1.08)	0.745
>0-2%	1.06 (1.01 ,1.12)	0.017	1.04 (0.98 ,1.11)	0.204	1.04 (0.98 ,1.11)	0.176
2.1-10%	1.08 (1.05 ,1.12)	0.000	1.02 (0.98 ,1.06)	0.331	1.01 (0.97 ,1.05)	0.606
10.1-20%	1.15 (1.1 ,1.21)	0.000	1.07 (1.01 ,1.13)	0.018	1.05 (0.99 ,1.11)	0.098
20.1-30%	1.2 (1.11 ,1.28)	0.000	1.08 (1 ,1.16)	0.059	1.03 (0.96 ,1.12)	0.377
30.1%-40	1.27 (1.14 ,1.41)	0.000	1.12 (1 ,1.26)	0.044	1.1 (0.97 ,1.23)	0.125
40.1%-	1.6 (1.45 ,1.78)	0.000	1.49 (1.33 ,1.68)	0.000	1.44 (1.28 ,1.61)	0.000
Hospital COVID-19 X Black						
Before pandemic	reference		reference		reference	
0%	1.01 (0.97 ,1.04)	0.746	1 (0.97 ,1.04)	0.99	1 (0.96 ,1.03)	0.939
>0-2%	1.04 (1.01 ,1.07)	0.021	1.02 (0.99 ,1.06)	0.218	1.03 (1 ,1.07)	0.056
2.1-10%	1.04 (1.02 ,1.06)	0.000	1 (0.98 ,1.02)	0.905	1.01 (0.99 ,1.03)	0.519
10.1-20%	1.02 (0.99 ,1.04)	0.142	0.98 (0.96 ,1.01)	0.186	0.99 (0.96 ,1.01)	0.320
20.1-30%	1.01 (0.98 ,1.05)	0.420	0.99 (0.95 ,1.03)	0.589	0.99 (0.95 ,1.03)	0.712
30.1%-40	1.05 (0.99 ,1.11)	0.139	1.05 (0.98 ,1.11)	0.173	1.05 (0.99 ,1.12)	0.131
40.1%-	1.2 (1.1 ,1.31)	0.000	1.2 (1.09 ,1.31)	0.000	1.21 (1.11 ,1.33)	0.000
Hospital COVID-19 X Hispanic						
Before pandemic	reference		reference		reference	
0%	1.15 (1.1 ,1.2)	0.006	1.15 (1.1 ,1.2)	0.000	1.05 (1.01 ,1.1)	0.010
>0-2%	1.06 (1.02 ,1.11)	0.000	1.05 (1.01 ,1.1)	0.020	1.06 (1.02 ,1.11)	0.008
2.1-10%	1.12 (1.1 ,1.15)	0.000	1.06 (1.03 ,1.09)	0.000	1.08 (1.05 ,1.11)	0.000
10.1-20%	1.2 (1.16 ,1.24)	0.000	1.09 (1.05 ,1.13)	0.000	1.13 (1.09 ,1.17)	0.000
20.1-30%	1.21 (1.16 ,1.26)	0.000	1.07 (1.02 ,1.12)	0.010	1.12 (1.07 ,1.17)	0.000
30.1%-40	1.28 (1.2 ,1.36)	0.000	1.11 (1.04 ,1.19)	0.001	1.17 (1.1 ,1.24)	0.000
40.1%-	1.6 (1.47 ,1.75)	0.000	1.38 (1.26 ,1.52)	0.000	1.45 (1.32 ,1.59)	0.000
Age						
65-69	reference		reference		reference	
70-74	1.02 (1.02 ,1.03)	0.000	1.11 (1.11 ,1.12)	0.000	1.11 (1.1 ,1.12)	0.000
75-79	1.16 (1.15 ,1.17)	0.000	1.27 (1.26 ,1.28)	0.000	1.27 (1.26 ,1.28)	0.000
80-84	1.34 (1.33 ,1.35)	0.000	1.5 (1.49 ,1.51)	0.000	1.5 (1.49 ,1.51)	0.000
85-89	1.6 (1.58 ,1.61)	0.000	1.82 (1.8 ,1.83)	0.000	1.82 (1.81 ,1.84)	0.000
90-94	1.94 (1.92 ,1.96)	0.000	2.27 (2.25 ,2.3)	0.000	2.29 (2.27 ,2.32)	0.000
95-	2.46 (2.43 ,2.49)	0.000	2.96 (2.92 ,3)	0.000	3 (2.96 ,3.04)	0.000
Sex						
Male	reference		reference		reference	
Female	0.91 (0.9 ,0.91)	0.000	0.98 (0.98 ,0.99)	0.000	0.98 (0.98 ,0.98)	0.000

© 2024 Glance LG et al. *JAMA Network Open*.

© 2024 Glance LG et al. *JAMA Network Open*.

Supplement Table 3. 30-day Mortality and Morbidity

	Unadusted		Patient Charactistics		Patient and Hospital Characteristics	
	OR (95% CI)	P	OR (95% CI)	P	OR (95% CI)	P
Admission source						
Community	reference		reference		reference	
Hospital	1.76 (1.72 ,1.8)	0.000	1.3 (1.28 ,1.33)	0.000	1.31 (1.29 ,1.34)	0.000
SNF/Nursing Home	1.73 (1.7 ,1.77)	0.000	1.44 (1.41 ,1.47)	0.000	1.45 (1.42 ,1.48)	0.000
Other	1.5 (1.45 ,1.55)	0.000	1.29 (1.26 ,1.33)	0.000	1.3 (1.26 ,1.33)	0.000
Dual-eligible	1 (0.99 ,1.01)	0.406	0.91 (0.9 ,0.92)	0.000	0.92 (0.91 ,0.92)	0.000
Medicare Advantage			0.98 (0.97 ,0.99)	0.002	0.99 (0.98 ,1)	0.040
Functional Status/frailty						
Malnutrition			1.29 (1.25 ,1.32)	0.000	1.31 (1.27 ,1.34)	0.000
Senility			1.25 (1.2 ,1.29)	0.000	1.23 (1.18 ,1.27)	0.000
Cognitive			1.17 (1.15 ,1.18)	0.000	1.16 (1.15 ,1.18)	0.000
Dependent on provider			1.38 (1.35 ,1.41)	0.000	1.33 (1.3 ,1.35)	0.000
Pre-existing Conditions						
COVID-19			2.02 (1.99 ,2.06)	0.000	2.02 (1.99 ,2.06)	0.000
Shock			3.2 (3.17 ,3.23)	0.000	3.19 (3.16 ,3.22)	0.000
Myocardial infarction						
No prior myocardial infarction			reference		reference	
Prior ST-segment elevation MI			3.52 (3.34 ,3.7)	0.000	3.51 (3.34 ,3.69)	0.000
Prior Non-ST-segment elevation MI			1.61 (1.59 ,1.63)	0.000	1.6 (1.58 ,1.62)	0.000
Prior other MI			1.17 (1.15 ,1.19)	0.000	1.18 (1.16 ,1.19)	0.000
Congestive heart failure						
None			reference		reference	
Systolic CHF			1.25 (1.24 ,1.26)	0.000	1.25 (1.24 ,1.26)	0.000
Diastolic CHF			0.99 (0.98 ,1)	0.007	0.99 (0.98 ,1)	0.014
Systolic and diastolic CHF			1.19 (1.18 ,1.21)	0.000	1.2 (1.18 ,1.22)	0.000
Unspecified CHF			1.29 (1.28 ,1.3)	0.000	1.28 (1.27 ,1.3)	0.000
Pulmonary						
Acute respiratory distress syndrome			5.63 (5.44 ,5.83)	0.000	5.7 (5.51 ,5.9)	0.000
Pulmonary edema			1.27 (1.24 ,1.3)	0.000	1.27 (1.24 ,1.3)	0.000
Pulmonary interstitial disease			1.38 (1.36 ,1.41)	0.000	1.39 (1.37 ,1.42)	0.000
Acute respiratory failure			1.76 (1.74 ,1.78)	0.000	1.77 (1.75 ,1.79)	0.000
Acute on chronic respiratory failure			1.68 (1.66 ,1.7)	0.000	1.69 (1.67 ,1.71)	0.000
Respiratory failure, unspecified			2.25 (2.19 ,2.31)	0.000	2.25 (2.2 ,2.31)	0.000
Elixhauser comorbidities						
Alcohol abuse			1.11 (1.1 ,1.13)	0.000	1.12 (1.11 ,1.14)	0.000
Lymphoma			1.36 (1.34 ,1.38)	0.000	1.37 (1.36 ,1.39)	0.000
Leukemia			1.67 (1.65 ,1.7)	0.000	1.69 (1.66 ,1.72)	0.000
Metastatic cancer			3.64 (3.59 ,3.69)	0.000	3.68 (3.63 ,3.73)	0.000
Solid tumor			1.58 (1.56 ,1.59)	0.000	1.58 (1.56 ,1.6)	0.000
Cerebrovascular disease			1.19 (1.18 ,1.21)	0.000	1.2 (1.18 ,1.21)	0.000
Coagulopathy			1.23 (1.22 ,1.24)	0.000	1.23 (1.22 ,1.24)	0.000
Dementia			1.33 (1.32 ,1.34)	0.000	1.33 (1.32 ,1.34)	0.000
Liver disease, mild			1.14 (1.13 ,1.16)	0.000	1.15 (1.14 ,1.17)	0.000
Liver disease and failure, moderate to severe			2.6 (2.56 ,2.65)	0.000	2.63 (2.59 ,2.68)	0.000
Chronic pulmonary disease			0.99 (0.98 ,1)	0.001	0.99 (0.98 ,0.99)	0.000
Neurologic disorder, other			1.32 (1.3 ,1.33)	0.000	1.32 (1.31 ,1.33)	0.000
Seizures and epilepsy			1.01 (0.99 ,1.02)	0.348	1 (0.99 ,1.01)	0.434
Paralysis			1.02 (1.01 ,1.04)	0.001	1.03 (1.02 ,1.05)	0.000
Peripheral vascular disease			1.06 (1.04 ,1.09)	0.000	1.1 (1.08 ,1.12)	0.000
Pulmonary circulation disease			1.22 (1.21 ,1.23)	0.000	1.22 (1.21 ,1.24)	0.000
Renal failure, moderate			1.13 (1.12 ,1.14)	0.000	1.14 (1.13 ,1.14)	0.000
Renal failure, severe			1.72 (1.71 ,1.74)	0.000	1.72 (1.7 ,1.74)	0.000
Peptic ulcer disease with bleeding			1.16 (1.14 ,1.18)	0.000	1.17 (1.15 ,1.19)	0.000
Valvular disease			1.13 (1.12 ,1.14)	0.000	1.13 (1.12 ,1.14)	0.000
Weight loss			1.43 (1.4 ,1.47)	0.000	1.43 (1.39 ,1.46)	0.000
Dialysis			1.02 (1 ,1.03)	0.029	1.02 (1 ,1.04)	0.029

© 2024 Glance LG et al. *JAMA Network Open*.

Supplement Table 3. 30-day Mortality and Morbidity						
	Unadusted		Patient Charactistics		Patient and Hospital Characteristics	
	OR (95% CI)	P	OR (95% CI)	P	OR (95% CI)	P
Prior procedures						
PCI			0.85 (0.85 ,0.86)	0.000	0.85 (0.84 ,0.86)	0.000
CABG			0.98 (0.97 ,0.99)	0.000	0.97 (0.96 ,0.98)	0.000
Heart valve surgery			0.86 (0.84 ,0.87)	0.000	0.86 (0.85 ,0.88)	0.000
Left ventricular asist device			1.18 (1.02 ,1.35)	0.023	1.21 (1.05 ,1.4)	0.008
Kidney transplant			0.65 (0.63 ,0.68)	0.000	0.67 (0.65 ,0.69)	0.000
Heart transplant			0.75 (0.68 ,0.82)	0.000	0.76 (0.7 ,0.83)	0.000
Liver transplant			0.32 (0.3 ,0.34)	0.000	0.32 (0.3 ,0.34)	0.000
Hospital characteristics						
ECMO availability					0.95 (0.92 ,0.98)	0.000
Bed size						
<= 50					reference	
51-149					0.97 (0.93 ,1.01)	0.168
150-249					1 (0.95 ,1.05)	0.933
250-499					1 (0.94 ,1.06)	0.937
500-					0.97 (0.9 ,1.04)	0.399
Average daily census						
<=20					reference	
21-50					1.01 (0.96 ,1.05)	0.717
51-150					1.05 (0.99 ,1.1)	0.078
151-300					1.07 (1.01 ,1.14)	0.025
301-					1.11 (1.03 ,1.19)	0.006
Resident-to-bed ratio						
0					reference	
>0-0.10					0.97 (0.95 ,1)	0.031
0.11-0.20					0.99 (0.96 ,1.02)	0.596
0.21-0.40					0.92 (0.89 ,0.95)	0.000
0.41-					0.91 (0.87 ,0.95)	0.000
Disproportionate share percentage (DSH)						
0-9.9%					reference	
10.0-24.9%					1.09 (1.05 ,1.14)	0.000
25.0-49.9%					1.16 (1.12 ,1.21)	0.000
50.0%-					1.16 (1.1 ,1.23)	0.000
Region						
New England					reference	
Middle Atlantic					1.05 (1 ,1.1)	0.055
South Atlantic					1.07 (1.02 ,1.11)	0.003
East North Central					1.01 (0.96 ,1.05)	0.808
East South Central					1.09 (1.04 ,1.14)	0.000
West North Central					0.95 (0.9 ,1)	0.033
West South Central					0.98 (0.93 ,1.02)	0.300
Mountain					0.87 (0.82 ,0.91)	0.000
Pacific					0.8 (0.77 ,0.84)	0.000
Puerto Rico					3 (2.52 ,3.57)	0.000

© 2024 Glance LG et al. *JAMA Network Open*.

Supplement Table 3. 30-day Mortality and Morbidity

	Unadusted		Patient Charactistics		Patient and Hospital Characteristics	
	OR (95% CI)	P	OR (95% CI)	P	OR (95% CI)	P
Time	0.997 (0.997 ,0.997)	0.000	0.996 (0.996 ,0.996)	0.000	0.996 (0.996 ,0.996)	0.000
Admission month						
January	reference	0.000	reference		reference	
February	0.97 (0.96 ,0.98)	0.000	0.98 (0.97 ,0.99)	0.000	0.98 (0.97 ,0.99)	0.000
March	0.96 (0.95 ,0.97)	0.000	0.99 (0.98 ,1)	0.084	0.99 (0.98 ,1)	0.018
April	0.96 (0.95 ,0.97)	0.000	0.97 (0.96 ,0.98)	0.000	0.96 (0.95 ,0.97)	0.000
May	0.9 (0.9 ,0.91)	0.000	0.93 (0.92 ,0.94)	0.000	0.93 (0.92 ,0.94)	0.000
June	0.88 (0.87 ,0.89)	0.000	0.92 (0.92 ,0.93)	0.000	0.92 (0.91 ,0.93)	0.000
July	0.89 (0.89 ,0.9)	0.000	0.94 (0.93 ,0.95)	0.000	0.94 (0.93 ,0.95)	0.000
August	0.91 (0.9 ,0.92)	0.000	0.95 (0.94 ,0.96)	0.000	0.95 (0.94 ,0.96)	0.000
September	0.92 (0.91 ,0.93)	0.000	0.96 (0.95 ,0.97)	0.000	0.96 (0.95 ,0.97)	0.000
October	0.94 (0.93 ,0.95)	0.000	0.98 (0.97 ,0.99)	0.001	0.98 (0.97 ,0.99)	0.000
November	0.95 (0.94 ,0.96)	0.000	0.98 (0.97 ,0.99)	0.000	0.98 (0.97 ,0.99)	0.000
December	0.92 (0.91 ,0.93)	0.000	0.98 (0.97 ,0.99)	0.000	0.98 (0.97 ,0.99)	0.001
Admission day of week						
Sunday	0.97 (0.97 ,0.98)	0.000	0.99 (0.98 ,0.99)	0.000	0.99 (0.98 ,0.99)	0.000
Monday	reference		reference		reference	
Tuesday	1.03 (1.02 ,1.04)	0.000	1.03 (1.02 ,1.04)	0.000	1.03 (1.02 ,1.04)	0.000
Wednesday	0.99 (0.99 ,1)	0.046	1 (0.99 ,1)	0.477	1 (0.99 ,1)	0.403
Thursday	0.98 (0.97 ,0.99)	0.000	0.99 (0.98 ,0.99)	0.001	0.99 (0.98 ,0.99)	0.001
Friday	0.98 (0.97 ,0.98)	0.000	0.99 (0.98 ,0.99)	0.000	0.99 (0.98 ,0.99)	0.000
Saturday	0.97 (0.96 ,0.98)	0.000	0.98 (0.98 ,0.99)	0.000	0.98 (0.98 ,0.99)	0.000

© 2024 Glance LG et al. *JAMA Network Open*.
