# Supplementary material for: Hospital Strain During the COVID-19 Pandemic and Outcomes in Older Racial and Ethnic Minority Adults
Source: JAMA Netw Open. 2024 Oct 15;7(10):e2438563. doi: 10.1001/jamanetworkopen.2024.38563 (PMC11581618; doi:10.1001/jamanetworkopen.2024.38563)
Supplement: Supplement 2. — Data Sharing Statement [file jamanetwopen-e2438563-s002.pdf]

Data Sharing Statement

Glance. Hospital Strain During the COVID-19 Pandemic and Outcomes in Older Racial and Ethnic Minority Adults. *JAMA Netw Open*. Published October 15, 2024.
doi:10.1001/jamanetworkopen.2024.38563

Data
Data available: No
